# Anisotropic longitudinal water proton relaxation in white matter investigated ex vivo in porcine spinal cord with sample rotation

**DOI:** 10.1038/s41598-024-63483-0

**Published:** 2024-06-05

**Authors:** Niklas Wallstein, André Pampel, Carsten Jäger, Roland Müller, Harald E. Möller

**Affiliations:** 1 NMR Methods & Development Group, Max Planck Institute for Human Cognitive and Brain Sciences, Leipzig, Germany; 2 Department of Neurophysics, Max Planck Institute for Human Cognitive and Brain Sciences, Leipzig, Germany; 3 Paul Flechsig Institute-Center of Neuropathology and Brain Research, Medical Faculty, Leipzig University, Leipzig, Germany; 4 Felix Bloch Institute for Solid State Physics, Leipzig University, Leipzig, Germany

**Keywords:** Anisotropy, Inversion recovery, Longitudinal relaxation, Magnetization transfer, MP2RAGE, Orientation dependence, Biophysics, Biomarkers, Physics

## Abstract

A variation of the longitudinal relaxation time $T_{1}$ in brain regions that differ in their main fiber direction has been occasionally reported, however, with inconsistent results. Goal of the present study was to clarify such inconsistencies, and the origin of potential $T_{1}$ orientation dependence, by applying direct sample rotation and comparing the results from different approaches to measure $T_{1}$. A section of fixed porcine spinal cord white matter was investigated at 3 T with variation of the fiber-to-field angle $\theta_{{{\text{FB}}}}$. The experiments included one-dimensional inversion-recovery, MP2RAGE, and variable flip-angle $T_{1}$ measurements at 22 °C and 36 °C as well as magnetization-transfer (MT) and diffusion-weighted acquisitions. Depending on the technique, different degrees of $T_{1}$ anisotropy (between 2 and 10%) were observed as well as different dependencies on $\theta_{{{\text{FB}}}}$ (monotonic variation or $T_{1}$ maximum at 30–40°). More pronounced anisotropy was obtained with techniques that are more sensitive to MT effects. Furthermore, strong correlations of $\theta_{{{\text{FB}}}}$-dependent MT saturation and $T_{1}$ were found. A comprehensive analysis based on the binary spin-bath model for MT revealed an interplay of several orientation-dependent parameters, including the transverse relaxation times of the macromolecular and the water pool as well as the longitudinal relaxation time of the macromolecular pool.

## Introduction

Differences in the longitudinal relaxation time $T_{1}$ of water protons in different tissue types are a key contrast mechanism in magnetic resonance imaging (MRI). A more recent aim is to use relaxation parameters for quantitative analyses and interpretation, in terms of tissue microstructure or myelination patterns, in particular in the brain or spinal cord ^1–7^. A detailed understanding of the underlying physical mechanisms is hampered by the remarkable variation in reported $T_{1}$ values in white matter (WM), dependent on the anatomical region of interest (ROI) ^8,9^, and also by the potential impact from different measurement techniques ^10,11^. This has led to attempts to obtain reproducible relaxation parameters independent of the acquisition strategy ^11–13^. On a more fundamental level, it is well established that the longitudinal relaxation in WM cannot be fully captured by a model with monoexponential recovery and a uniform $T_{1}$, although this is often an underlying assumption ^2,5,13^. Since an important contribution to water proton relaxation stems from cross-relaxation with semi-solid membrane components and macromolecules, comprehensive modeling necessitates consideration of multiple proton pools and magnetization transfer (MT) between them ^2,13,14^.

Recent work has further drawn attention to anisotropic longitudinal relaxation characteristics in human WM in vivo ^15–18^ as well as in *ex-vivo* tissue samples with a high degree of structural order ^17,19,20^. This is remarkable because no anisotropy of longitudinal relaxation or MT was found in early investigations ^21^. However, inconsistencies also exist between studies reporting orientation dependence. Kauppinen et al. ^15–17^ obtained $T_{1}$ anisotropy at 3 T for human WM fibers, with a $T_{1}$ maximum at a fiber-to-field angle $\theta_{{{\text{FB}}}}$≈ 50° and an orientation-dependent variation, of about 7%. Efforts to reproduce and elucidate this trend ex vivo, in rat brain at 9.4 T, with other acquisition techniques were only partially successful and showed a different orientation dependence ^17^. Other ex vivo measurements at the same field strength showed no observable $T_{1}$ anisotropy with inversion recovery, but a high anisotropy of the transverse relaxation time $T_{2}$ ^19,20^. Using a two-point measurement, Schyboll et al. ^18^ observed a monotonically increasing longitudinal relaxation rate $R_{1}$ (i.e., decreasing $T_{1}$) by approximately 3% upon increasing $\theta_{{{\text{FB}}}}$ from 0° to 90° in human WM. They assumed that this orientation effect at 3 T was caused by differences in dipolar relaxation ^22,23^, due to the anisotropic molecular environment within the myelin sheaths, rather than from relaxation induced by susceptibility differences ^24^.

Considering the residual dipolar couplings between motion-restricted protons in microstructurally ordered cylindrical nerve fibers and their enveloping myelin sheath, anisotropic properties are expected rather than surprising. For example, orientation dependence has been reported for the transverse relaxation time $T_{2}^{B}$ of semi-solid myelin membrane components in MT experiments ^25^.

Although the accuracy of the aforementioned studies is not questioned, there are limitations, for example, related to scanner hardware and imaging protocols. In particular, $$T_{1}$$ anisotropy was assessed by comparing regions with different $$\theta_{{{\text{FB}}}}$$ in *in-vivo* human studies. Inversion times (TIs) were restricted to a narrow range due to tolerable scan time in vivo and the need to integrate the imaging readout. Moreover, only soft inversion pulses (durations $$\tau_{p}$$ > 100 μs$$)$$ were used, which do not achieve well-defined initial conditions for the semi-solid proton pool. Finally, gradient-recalled-echo (GRE) sequences employ a considerable number of readout pulses leading to MT effects ^26^, which are typically not accounted for in the analysis ^27,28^.

The considerable discrepancies between previous studies show that a sufficient understanding of anisotropic relaxation has not yet been achieved. An initial goal of the current study was, therefore, to establish carefully controlled experimental conditions for precise and reproducible investigations of orientation-dependent $T_{1}$ in WM. This included experiments at different temperatures to address one aspect of limited comparability between ex vivo MRI in fixed tissue, which is typically performed at room temperature, and in vivo MRI at physiological temperature. In addition, it allows the clarification of whether a consistent anisotropic behavior can already be observed at room temperature. Our main hypothesis was that $T_{1}$ anisotropy results from an interplay of orientation-dependent longitudinal relaxation of non-aqueous protons and MT with water protons. Neglecting these effects holds the potential of misleading interpretation in experiments aiming at the quantification of $T_{1}$ due to inherently limited accuracy. We further hypothesized that different strategies for $T_{1}$ measurements may show distinct differences in their sensitivity to orientation effects. This is based on the reported differences in the variation of *T*_1_ with $\theta_{{{\text{FB}}}}$ in previous in vivo studies utilizing Magnetization-Prepared 2 Rapid Acquisition Gradient Echoes (MP2RAGE) ^28^ protocols and two-point *T*_1_ measurements. In addition, orientation-dependent MT weighting caused by small flip-angle readout pulses, in these aforementioned in vivo GRE acquisitions, might have contributed to a variable extent to the results. To investigate these hypotheses we compared different $T_{1}$ measurement techniques, including the gold standard classical inversion recovery (IR) ^29^ as well as GRE approaches with variable flip angles (VFA) ^30,31^ and MP2RAGE, which are frequently employed in brain MRI studies. Differences between these methods include: different dynamic ranges for measuring the recovery of longitudinal magnetization, different initial magnetization states, especially for non-aqueous protons, and different sensitivities to MT processes between aqueous and non-aqueous proton pools. Another aim was to isolate ‘true’ orientation effects by directly reorienting samples of ex vivo WM using a tiltable coil. This excludes confounding factors, such as differences in myelination or in distribution of fibers within a voxel, which may limit investigations that compare different brain regions with supposedly different main fiber orientations or fiber dispersions ^32^. In particular, we used a small segment of well-oriented porcine spinal cord WM. This allowed one-dimensional (1D) acquisitions along the main fiber orientation in order to consistently investigate a large number of experimental conditions in a reasonable scan time, including measurements at very short TIs.

## Materials and methods

### Sample preparation

**Figure 1:**
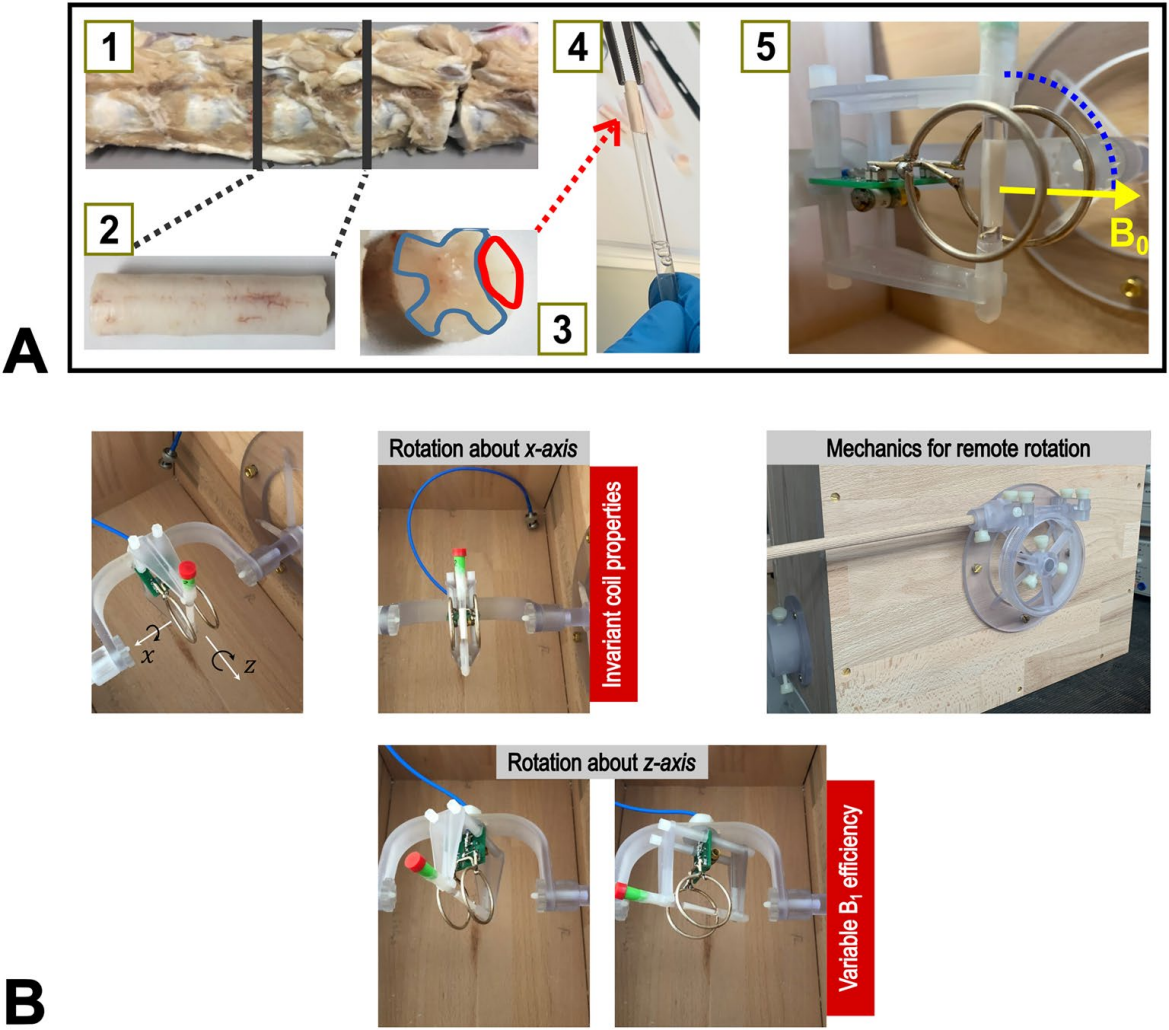
(**A**) Fresh pieces of porcine spinal column from a local slaughterhouse (*post mortem* interval 2–3 h) were fixated in 4% PFA/PBS solution *(1)*. A piece of spinal cord from segment L5/6 (lumbar vertebrae) was carefully dissected *(2)* and a part of the WM region (highlighted in red) *(3)* was placed in an NMR tube embedded in Fomblin *(4)*. Finally, the tube was placed in the central region of a Helmholtz coil. The setup allows remote reorientation of the coil and sample around the cylindrical axis of the coil, which runs through the isocenter of the magnet *(5)*. Therefore, the transmit and receive efficiency of the coil are invariant for acquisitions at different orientations. (**B**) Illustration of the possibilities for rotating the coil and sample about two axes ($x$- and $z$-axis). In the present study, only rotations about the $x$-axis were performed. These are carried out with constant coil properties (i.e., invariant transmit field and receive sensitivity).

A small section of porcine spinal cord WM was used for all experiments. Pieces of porcine spine were obtained from a local slaughterhouse within 2–3 h of death, immediately fixated in 4% paraformaldehyde (PFA) in phosphate buffered saline (PBS) at pH 7.4, and stored at 4 °C. After 5 days, the fixative was replaced with a fresh batch. After 6 weeks of fixation the samples were transferred into PBS with 0.1% sodium azide. From the lumbar segment (L5-6) the spinal cord was extracted and stored in PBS/NaN_3_. A small, largely homogeneous section of WM of the posterior funiculus (length 17 mm, diameter ≤ 4 mm) was excised from the center of the vertebra, where no nerve roots extended laterally, and washed in PBS for several hours. Subsequently, it was placed in a 5 mm NMR glass tube (Wilmad®, Wilmad-Labglass, NJ, USA) and embedded in Fomblin® (perfluoropolyether, Solvay Solexis, Bollate, Italy) to improve the susceptibility matching for the MRI measurements (Fig. 1).

Besides experiments in spinal cord WM, a sample of water doped with MnCl_2_ was used for sequence testing. The concentration was 0.135 mM to produce a $T_{1}$ of approximately 750 ms and a transverse relaxation time $T_{2}$ of approximately 50 ms at room temperature ^33^.

### Hardware setup

The NMR tube was placed in the center region of a custom-made TxRx Helmholtz coil (radius and spacing 16 mm) described elsewhere ^34^. This coil was mounted inside a cuboid box (235 mm × 295 mm × 380 mm; Supplementary Fig. S1) made of 18 mm-thick beech wood panels to enable measurements above room temperature ^35^. Elevated temperature was achieved by a stream of heated air from a heat exchanger located outside the magnet room. The airflow was transferred through approximately 12 m of flexible tubing (inner diameter 35 mm), which was thermally insulated with polyurethane foam. It was drawn from the magnet room via a waveguide by an adjustable vacuum cleaner (WD 3P, Kärcher, Winnenden, Germany), passed through a heater, and then transferred into the box via another waveguide. The heater consisted of a simple heating plate and an aluminum heat sink encased in sheet metal as a heat exchanger. To avoid hysteresis effects of the heater, which would result in periodic temperature fluctuations, the heating plate’s bimetal regulator was set to maximum and both the vacuum cleaner and the heater were adjustable via external phase-angle controllers. Temperature was monitored at three positions: inside the box close to the sample, in the scanner room, and inside the tubing (Supplementary Fig. S2), using a fiberoptic system (Optocon; Weidmann, Dresden, Germany). Another feature of this setup was a mechanism that allowed precise sample reorientation without removing the box from the scanner’s isocenter. For this purpose, a 3D-printed universal joint, combined with a worm gear, was mounted on the outside of the wooden box to transfer the rotation of a wooden crank (one full rotation resulted in a 2° tilt of the NMR tube), which was easily accessible to the operator.

### MR-investigations

All experiments were performed on a MAGNETOM Skyra^fit^ 3 T scanner (Siemens Healthineers, Erlangen, Germany) operated under the software baseline *syngo* MR VE11E. For all measurements, customized pulse sequences were utilized with an identical 1D readout (0.965 mm nominal resolution) along the sample axis. During the experiments (total duration 10–12 h for each temperature), the temperature inside the box was maintained at 35.7–36.2 °C or at 21.6–22.9 °C to minimize confounds due to temperature fluctuations or drifts (Supplementary Fig. S2). The reference transmitter amplitude for a 1 ms-long 180° rectangular pulse was carefully calibrated (17.3 V) with a pulse-width array experiment ^34,36^. It consisted of 20 measurements using rectangular pulses with stepwise incremented durations $$($$1.7 ms ≤ $$\tau_{p}$$ ≤ 2.27 ms$$)$$.

The sample orientation was varied between angles of 0° (corresponding to a parallel alignment with the external field ${\mathbf{B}}_{0}$) and 90° (corresponding to a perpendicular alignment with the external field ${\mathbf{B}}_{0}$) in a pseudorandomized order without removing the box from the magnet. Measurements were performed with a total of 11 rotation angles, starting and ending with a 90° orientation, to verify stable conditions (e.g., absence of scanner drifts, no tissue degradation). At selected rotation angles (0°, 40°, 90°), $\theta_{{{\text{FB}}}}$ was validated by diffusion tensor imaging (DTI; echo time, TE = 80 ms; repetition time, TR = 4 s; acquisition time, TA = 4 min 28 s) with 60 gradient directions (two different gradient strengths with $b$-values of 1200 and 1500 s/mm^2^) interleaved with 7 measurements without diffusion-sensitizing gradients. $\theta_{{{\text{FB}}}}$ was retrieved from the angle between the diffusion tensor eigenvector corresponding to the largest eigenvalue (principal eigenvector), ${\mathbf{e}}_{1}$, and the direction of ${\mathbf{B}}_{0}$ according to:

1 $$\cos \theta_{FB} = {\mathbf{e}}_{1} \cdot {\mathbf{B}}_{0} /\left| {{\mathbf{e}}_{1} } \right|\left| {{\mathbf{B}}_{0} } \right|$$

The local transmit field $$B_{1}^{ + }$$ was measured with a flip-angle array of rectangular pulses (nominal $$\alpha$$ = 0°, 45°, 90°, …, 315°, TR = 4 s, $${\uptau }_{p}$$ = 500 μs$$)$$ by fitting the magnitude signal to $${\text{abs}}\left( {\sigma \cdot {\text{sin}}\left( {x_{{{\text{scale}}}} \alpha } \right)} \right)$$, where $$\sigma$$ is a scaling factor that accounts for differences in local proton density and coil sensitivity, and $$x_{{{\text{scale}}}}$$ is the local $$B_{1}^{ + }$$ scaling factor.

The methods for measuring $T_{1}$ included IR, MP2RAGE, and VFA, which all employed 1D projections (i.e., without slice selection and phase encoding). IR experiments were performed with both hard, rectangular ($\tau_{p}$ = 40 μs) and soft, adiabatic inversion pulses (BIR-4, $\tau_{p}$ = 5 ms) ^37^ to create different initial conditions, in particular of proton magnetization associated with motion-restricted macromolecules or membranes ^2,13^. Datasets consisting of 23 logarithmically spaced inversion times (770 μs ≤ TI ≤ 10 s, TR = 13 s) were acquired for both cases.

An alternative gradient spoiling strategy was implemented for 1D MP2RAGE imaging, with decreasing amplitudes and two alternating directions orthogonal to the readout direction to reduce confounding signal contributions from stimulated echoes (Supplementary Fig. S3). Additional radiofrequency (RF) spoiling ^38,39^ was performed with a 50° phase increment. Due to the restriction to 1D spatial encoding, the gradient schemes of the first (inner loop) and second (outer loop) phase-encoding directions were omitted, whereas all RF pulses (non-selective, rectangular shape; flip angle $\alpha$) of the GRE kernels were applied in order to obtain the same evolution of the longitudinal magnetization as in standard 3D MP2RAGE. Therefore, all $N_{{{\text{rep}}}}$ steps of the outer loop were identical and corresponded to simple sequence repetitions. Another feature was that a single readout per TI was sufficient for the 1D reconstruction and data analysis. This allowed the replacement of one $\alpha$-pulse of the GRE kernel by a 90° pulse of the same duration (100 μs) to improve the signal intensity. This 90° readout pulse was integrated in the last repetition ($N_{{{\text{rep}}}}$) for the first, and in repetition $N_{{{\text{rep}}}}$/2 for the second, TI at positions in the GRE kernel corresponding to the acquisition of the k-space center in 3D MP2RAGE (Supplementary Fig. S3). This strategy of placing the 90° pulses as a function of $N_{{{\text{rep}}}}$ was chosen to maximize the temporal distance between them so that the steady state was minimally perturbed. A choice of $N_{{{\text{rep}}}}$ = 20 ensured steady-state conditions at the time of these signal readouts. Further sequence parameters are summarized in Supplementary Table 1. Thirteen pairs of inversion times were acquired in separate scans. Some of the TI-values were adopted from Kauppinen et al. ^17^ to allow a direct comparison of the results.

VFA experiments consisted of 11 acquisitions with $\alpha$ ranging from 4° to 60° (Supplementary Table 1) using an identical spoiling strategy as for MP2RAGE. Additional two-point calculations of $T_{1}$ were also performed using parameters from a multi-parameter mapping (MPM) protocol (“proton density-weighted”: $\alpha$ = 4°; “$T_{1}$-weighted”: $\alpha$ = 25°) ^40,41^. Other sequence parameters are also included in Supplementary Table 1. Subtle variations (< 2%) of the local transmit field were accounted for by appropriate rescaling of the flip angles.

Finally, MT measurements were performed with a train of 250 Gaussian RF pulses ($\tau_{p}$ = 2 ms, amplitude ${\upgamma }B_{{1,{\text{RMS}}}}^{ + } /\left( {2{\uppi }} \right)$ = 500 Hz or 705 Hz, offset frequency 10 kHz; ${\upgamma }$ is the gyromagnetic ratio and $B_{{1,{\text{RMS}}}}^{ + }$ the root-mean-squared amplitude of the pulse) followed by a GRE readout (same as used for VFA experiments). Dual-sided saturation was achieved by alternating the offset frequency of subsequent RF events and by cosine modulation of the RF pulse shapes (inter-pulse delay of 250 μs) in separate experiments. The RF amplitude was scaled by an additional factor of 2 for cosine modulated pulses.

### Data analysis

The raw data obtained from the scanner were pre-processed using routines developed in-house in MATLAB (R2020b; MathWorks, Natick, MA, USA). The acquired data were multiplied by a Tukey windowing function ($\alpha$ = 0.5) ^42^, Fourier-transformed and phase corrected. Fitting was performed with real signals for IR experiments and with magnitude signals for all other measurements. Non-linear regression used the function “*nlinfit”*. The uncertainties of the determined $T_{1}$ values were specified as 95% confidence intervals using the “*nlparci”* algorithms of MATLAB’s Statistics and Machine Learning Toolbox, which are roughly equivalent to two standard deviations (SDs) obtained from the covariance matrix.

Two general approaches were used to analyze the data. First, fitting was performed according to analytical expressions for the specific measurement protocols:

IR-experiments were fitted assuming a monoexponential recovery of the signal according to:

2 $$S{\text{(TI)}} = \sigma \cdot \left( {1 - x_{{{\text{inv}}}} \exp \left( { - {\text{TI}}/T_{1} } \right)} \right),$$

where $x_{{{\text{inv}}}}$ is the inversion efficiency of the RF pulse and $\sigma$ is a scaling factor.

VFA $T_{1}$ measurements were fitted to the Ernst equation^43^, which is valid after the establishment of a (periodic) steady state:

3 $$S\left( \alpha \right) = \sigma \frac{{1 - {\text{exp}}\left( { - {\text{TR}}/T_{1} } \right)}}{{1 - {\text{exp}}\left( { - {\text{TR}}/T_{1} } \right){\text{cos}}\alpha }}\sin \alpha .$$

MP2RAGE data were analyzed assuming analytical expressions derived elsewhere ^27,28^, that is, the $z$-magnetization after the application of $n$ readout pulses of flip angle $\alpha$ is described by:

4 $$\begin{aligned} M_{{z,{\text{nrf}}}} \left( {M_{z} \left( 0 \right),T_{1} ,n,{\text{TR}},\alpha } \right) & = M_{z} \left( 0 \right)\left( {\cos\alpha \exp\left( { - {\text{TR}}/T_{1} } \right)} \right)^{n} \\ & \;\;\; + M_{0} \left( {1 - \exp\left( { - {\text{TR}}/T_{1} } \right)} \right)\frac{{1 - \left( {\cos\alpha \exp\left( { - {\text{TR}}/T_{1} } \right)} \right)^{n} }}{{1 - \cos\alpha \exp\left( { - {\text{TR}}/T_{1} } \right)}}. \\ \end{aligned}$$

During the period ${\Delta }t$ between the two acquisitions blocks, no RF pulses are applied, and the $z$-magnetization relaxes according to:

5 $$M_{{z,{\text{0rf}}}} \left( {M_{z} \left( 0 \right),T_{1} ,{\Delta }t} \right) = M_{z} \left( 0 \right){\text{exp}}\left( { - {\Delta }t/T_{1} } \right) + M_{0} \left( {1 - {\text{exp}}\left( { - {\Delta }t/T_{1} } \right)} \right).$$

The calculation of $T_{1}$ was not based on a library considering only two TIs, as by Marques et al. ^28^, but as described by Knight et al. ^15^ to be consistent with recent publications ^16,17^. We note that Eqs. (2–5) neglect MT and relaxation during the RF pulses.

The MT effect was characterized by the ratio of the water signal measured with the application of the MT pulse, $S_{{{\text{MT}}}}$, and in the absence of saturation, $S_{0}$, which is related to the conventional MT ratio (MTR) by

6 $${\text{MT}}_{{{\text{sat}}}} = \frac{{S_{{{\text{MT}}}} }}{{S_{0} }} = 1 - {\text{MTR}}.$$

Note that this empirical definition of “MT saturation” deviates from previously published relations that also consider confounding contributions to the MT effect ^44^.

It is obvious that this analysis, based on analytical expressions, is a simplification. In particular, a mono-exponential relaxation for IR experiments is not to be expected considering the heterogeneity of the proton environment in WM. To investigate these limitations, subsets of the IR curves covering different ranges of TIs were also analyzed. Building on this, more sophisticated approaches were used to try to explain differences in *T*_1_ estimations obtained for distinct subsets. These further analyses included simulations and parameter estimations based on an established matrix-algebra approach ^26^ and the binary spin-bath (BSB) model ^45–47^ with a free (liquid) water pool *A* and a macromolecular (semisolid) pool *B*. This explicitly considered relaxation during RF pulse application (relevant for the semisolid pool) and MT between the pools. Here, only the two types of IR experiments (hard-pulse and adiabatic-pulse inversion) were included and combined for simultaneous fitting (total of 2 × 23 × 11 = 506 data points for 11 orientations). The entire pulse sequences were simulated, assuming perfect spoiling of transverse magnetization during evolution periods. Transverse magnetization of the semi-solid pool *B* was neglected due to its rapid decay ^45,48,49^. Briefly, the propagation of the magnetization during the pulse sequence was computed by splitting the sequence into $n$ episodes with constant parameters, which may be either time delays or increments of the digitized RF pulses (see Supplementary Methods for details). Mathematically, this was achieved by sequentially multiplying the magnetization vector by a suitable propagator ^26,50,51^.

The BSB model describes the effect of an RF pulse on the magnetization of the semi-solid pool *B* solely as a saturation of $M_{z}^{B}$, which is captured by a suitable lineshape function ^13,45–47^. It is valid for relatively weak pulses as typically employed in clinical MT experiments. However, it is inappropriate for the IR sequence with an on-resonance hard pulse of 40 µs duration, which efficiently inverts $M_{z}^{A}$ and partially also $M_{z}^{B}$. To account for these conditions, the matrix element $P_{5,5}$ of the propagator (see Supplementary Methods, Eqs. S3–S5) describing the inversion pulse (both hard or adiabatic) was directly fitted without consideration of a specific lineshape or linewidth (expressed through $T_{2}^{B}$). This is equivalent to fitting $M_{z}^{B}$ directly after the inversion pulse, which we denote as $M_{z}^{B} \left( {0^{ + } } \right)$. To account for the different pulses (hard/adiabatic) and their durations (40 µs/5 ms), which impact the efficiency, $M_{z}^{B} \left( {0^{ + } } \right)$ was fitted separately for both types of inversion pulse. In summary, the experimental and simulated data were individually normalized to the value obtained for the longest TI, and the following parameters were fitted: the macromolecular proton fraction ${\text{MPF}} = M_{0}^{B} /\left( {M_{0}^{A} + M_{0}^{B} } \right)$, the relaxation times $T_{1}^{A}$, $T_{1}^{B}$ and $T_{2}^{A}$, and an MT rate constant $k$. Parameters that were considered to depend on orientation were adjusted separately for each rotation angle.

To obtain a first estimate of the spin-system parameters, both IR datasets from all orientations were simultaneously adjusted without consideration of anisotropy. The obtained parameters were then used as starting values for more complex analyses considering orientation dependence. As an exception, MPF was fixed to the initial (isotropic) result because the pool sizes should not depend on $\theta_{{{\text{FB}}}}$. Fixing MPF achieved reductions of parameter correlations in the fits. We further assumed that *k* and $T_{1}^{A}$ are invariant upon reorientation ^21^. They were, however, not fixed to their initial values but optimized as orientation-independent parameters. For all other parameters, different contributions from anisotropy were successively included in the fitting routine. In model *(1)*, only the influence of RF pulses on the semi-solid pool *B* was adapted independently for each orientation, that is, $M_{z}^{B} \left( {0^{ + } } \right)$ varies with $\theta_{{{\text{FB}}}}$. Model* (2)* additionally assumed orientation-dependence of the transverse relaxation time $T_{2}^{A}$. Finally, model *(3)* also assumed orientation dependence of the longitudinal relaxation time $T_{1}^{B}$ in addition to model *(2)*.

## Results

All experiments and analyses were first tested with the doped water sample to verify the accuracy of the measurements (e.g., appropriate timing, sufficient spoiling), fitting procedures, and susceptibility to system imperfections. These tests showed excellent agreement of the estimated $T_{1}$ with gold-standard IR measurements for all techniques (Supplementary Fig. S4 and Supplementary Table S2) and also stable results upon sample rotation (variations of $T_{1}$ estimates < 0.5%, Supplementary Fig. S5).

The fractional anisotropy (FA) obtained with DTI indicated a high anisotropy in the WM sample (i.e., well aligned fibers with relatively low orientation dispersion; see Supplementary Fig. S6). The results did not significantly change when repeating the measurements after 24 h at room temperature (Supplementary Fig. S7). Two ROIs (4 voxels each) characterized by FA > 0.65 were selected for further analyses based on the DTI results and their homogeneity with respect to high FA values, indicating a relatively low degree of fiber dispersion. As expected, the mean diffusivity (MD) was increased at higher temperature (≈ 0.4 × 10^–3^ mm^2^/s at 36 °C vs. ≈ 0.3 × 10^–3^ mm^2^/s at 22 °C, Supplementary Figs. S6c and S7c) but still smaller than previously reported in vivo values for human spinal cord (0.9 × 10^–3^ mm^2^/s) ^52^. The calculated $\theta_{{{\text{FB}}}}$ (Supplementary Figs. S6b and S7b), which describes the main fiber direction, indicated only minimal deviations (< 2°) from the adjusted rotation angle of the NMR tube.

The shape of the 1D $B_{1}^{ + }$ profile along the sample axis was almost independent of the orientation (see Supplementary Fig. S8). A subtle overall shift of the mean $B_{1}^{ + }$ was observed in comparisons of different rotation angles, however, with a negligible magnitude (< 1%) compared to the position-dependent profile.

In all orientation-dependent experiments, the results from the first and last acquisition at 90° rotation agreed within the experimental accuracy for all analyses. Thus, we can rule out that tissue degradation over time affected the observed orientation dependencies.

### Analyses assuming a uniform ${\varvec{T}}_{1}$ (fast-exchange limit)

#### Inversion-recovery experiments

**Figure 2:**
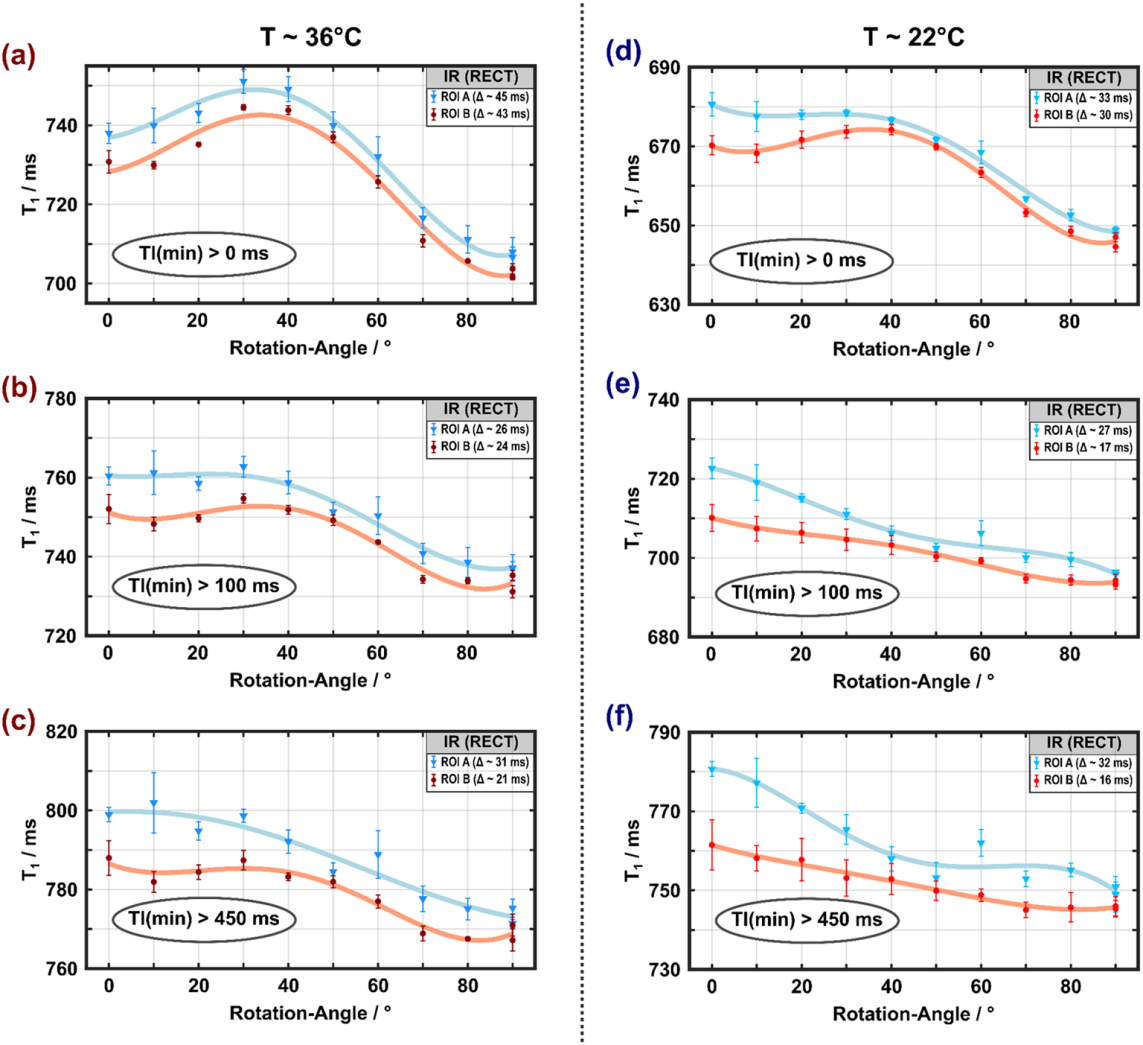
Apparent spin–lattice relaxation times obtained from monoexponential fitting of IR curves measured with hard-pulse inversion ($\tau_{p}$ = 40 µs) at 36 °C (**a**–**c**) and 22 °C (**d**–**f**) as a function of the rotation angle. Results from the two ROIs indicated by blue and red shading in Supplementary Fig. S5 are shown in matching colors. A $T_{1}$ maximum at rotation angles between 30° and 40° is visible if all inversion times are included in the (monoexponential) fits (**a**, **d**), especially for the higher temperature. It gradually disappears if only data with TI > 100 ms (**b**, **e**) or TI > 450 ms (**c**, **f**) are considered, yielding a trend of decreasing $T_{1}$ with increasing $\theta_{{{\text{FB}}}}$. Error bars indicated SDs over the ROIs.

**Figure 3:**
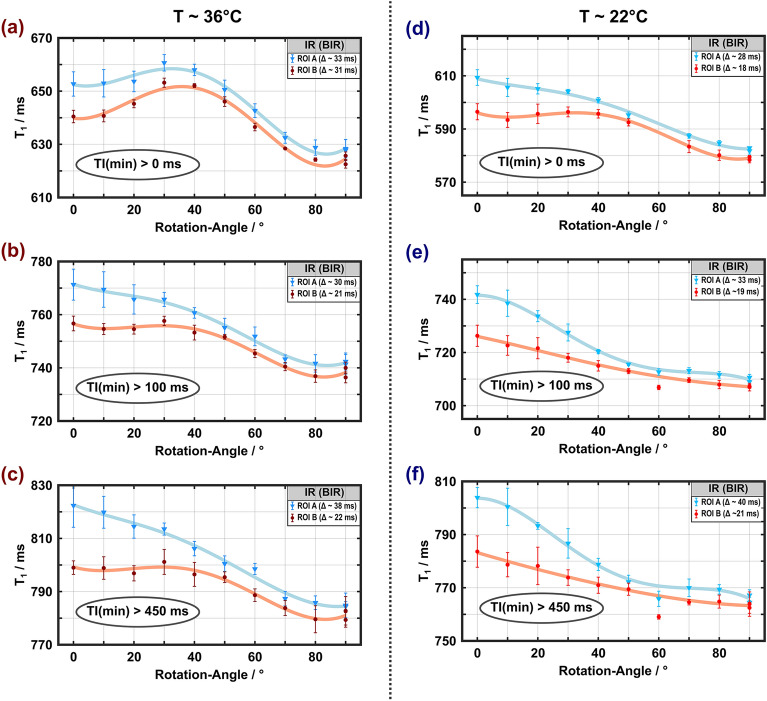
Apparent spin–lattice relaxation times obtained from monoexponential fitting of IR curves measured with adiabatic inversion ($\tau_{p}$ = 5 ms) at 36 °C (**a**–**c**) and 22 °C (**d**–**f**) as a function of the rotation angle (symbols, lines and ROI definitions as in Fig. 2). As in Fig. 2, a $T_{1}$ maximum at rotation angles between 30° and 40° is visible when all inversion times are included in the (monoexponential) fits (**a**, **d**), which is more pronounced for the higher temperature and disappears if only data with TI > 100 ms (**b**, **e**) or TI > 450 ms (**c**, **f**) are considered. Error bars indicated SDs over the ROIs. The datapoint at $\theta_{{{\text{FB}}}}$≈ 60° and 22 °C was an outlier due to incorrect frequency adjustment.

The IR data showed systematic deviations from a monoexponential recovery, which was particularly noticeable at short TI (TI < 100 ms, Supplementary Fig. S9). Analyzing the experiments under the simplifying assumption of Eq. (2) resulted in apparent $T_{1}$ values ranging from 710 to 800 ms and 620–820 ms at 36 °C for the hard-pulse inversion (Fig. 2) and the adiabatic inversion (Fig. 3), respectively. Measurements at room temperature resulted in consistently lower $T_{1}$ values than those at 36 °C, especially when all TIs were included in the analysis. At 36 °C, and somewhat less evident at 22 °C, a $T_{1}$ maximum appeared for both types of inversion at $\theta_{{{\text{FB}}}}$ between 30° and 40°, when all data points were included in the fits. Notably, these fitting approaches yielded substantially larger residuals (Supplementary Table S3) compared to results obtained when excluding data points acquired at very short TI. Consistent with observations by others ^13^, larger deviations were observed for the adiabatic inversion compared to the hard-pulse inversion. The dependence of the apparent $T_{1}$ on $\theta_{{{\text{FB}}}}$ changed towards a monotonically decaying function when only longer TIs were considered (TI > 100 ms or TI > 450 ms), and became more similar across temperatures and types inversion (Figs. 2b, c and 3b, c). However, the $T_{1}$ differences between $\theta_{{{\text{FB}}}}$ = 0° and 90° were small overall, on the order of 3–5%.

#### MP2RAGE experiments

**Figure 4:**
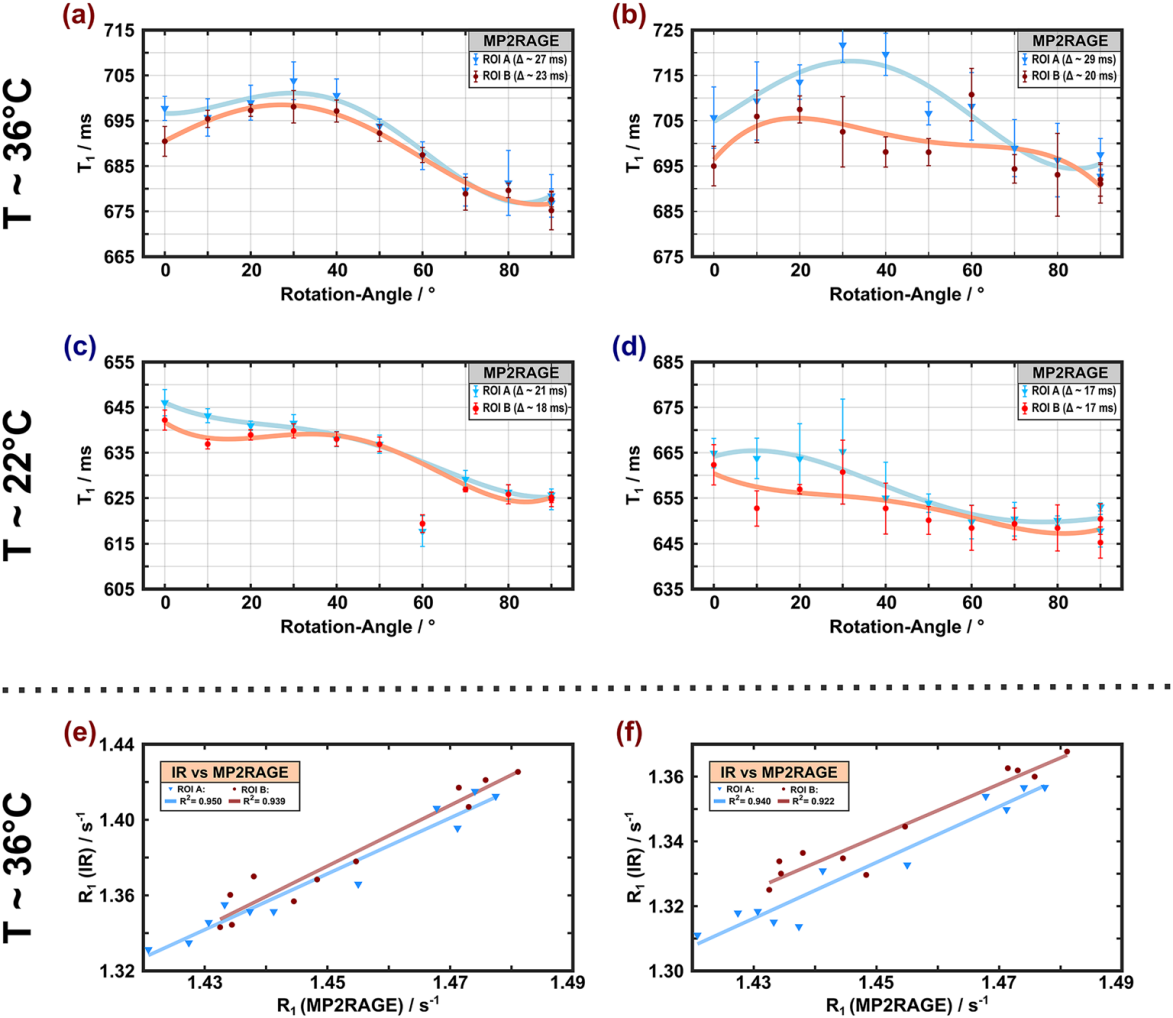
Apparent spin–lattice relaxation times obtained with MP2RAGE at 36 °C (**a**, **b**) and 22 °C (**c**, **d**) as a function of the rotation angle (symbols, lines and ROI definitions as in Fig. 2). The left column (**a**, **c**) shows results obtained upon considering all 13 pairs of TIs in the analysis whereas the right column (**b**, **d**) shows results obtained with a subset of only three pairs of TIs. A $T_{1}$ maximum at rotation angles between 30° and 40° is visible at 36 °C. As in Fig. 3, the datapoint at $\theta_{{{\text{FB}}}}$ = 60° and 22 °C was an outlier. Remarkably, high correlations of $\theta_{{{\text{FB}}}}$-dependent $R_{1}$ estimates obtained with MP2RAGE and IR are evident for both ROIs (**e**, **f**). The squared Pearson coefficients $R^{2}$ were slightly higher for the IR results obtained with fits to all TI-values (**e**) compared to the results obtained with TI > 100 ms (**f)**.

The $T_{1}$ values measured with MP2RAGE also exhibited a maximum around $\theta_{{{\text{FB}}}}$ between 30° and 40° at 36 °C (Fig. 4a, b) and a trend of a $T_{1}$ decay with increasing $\theta_{FB}$ at 22 °C, similar to the IR results obtained with longer TIs (Fig. 4c, d). The $T_{1}$ variations were relatively small (3–4%) across temperatures. Restricting the analysis to a subset of only three TI pairs ([300, 900] ms, [200, 1200] ms, [600, 1500] ms) that had been used in recent in vivo studies ^17^, resulted in wider confidence intervals (Supplementary Table S4). With the reduced set, the range of $T_{1}$ variations were slightly increased and the overall trends appeared less clear (Fig. 4b, d).

#### VFA and MPM experiments

**Figure 5:**
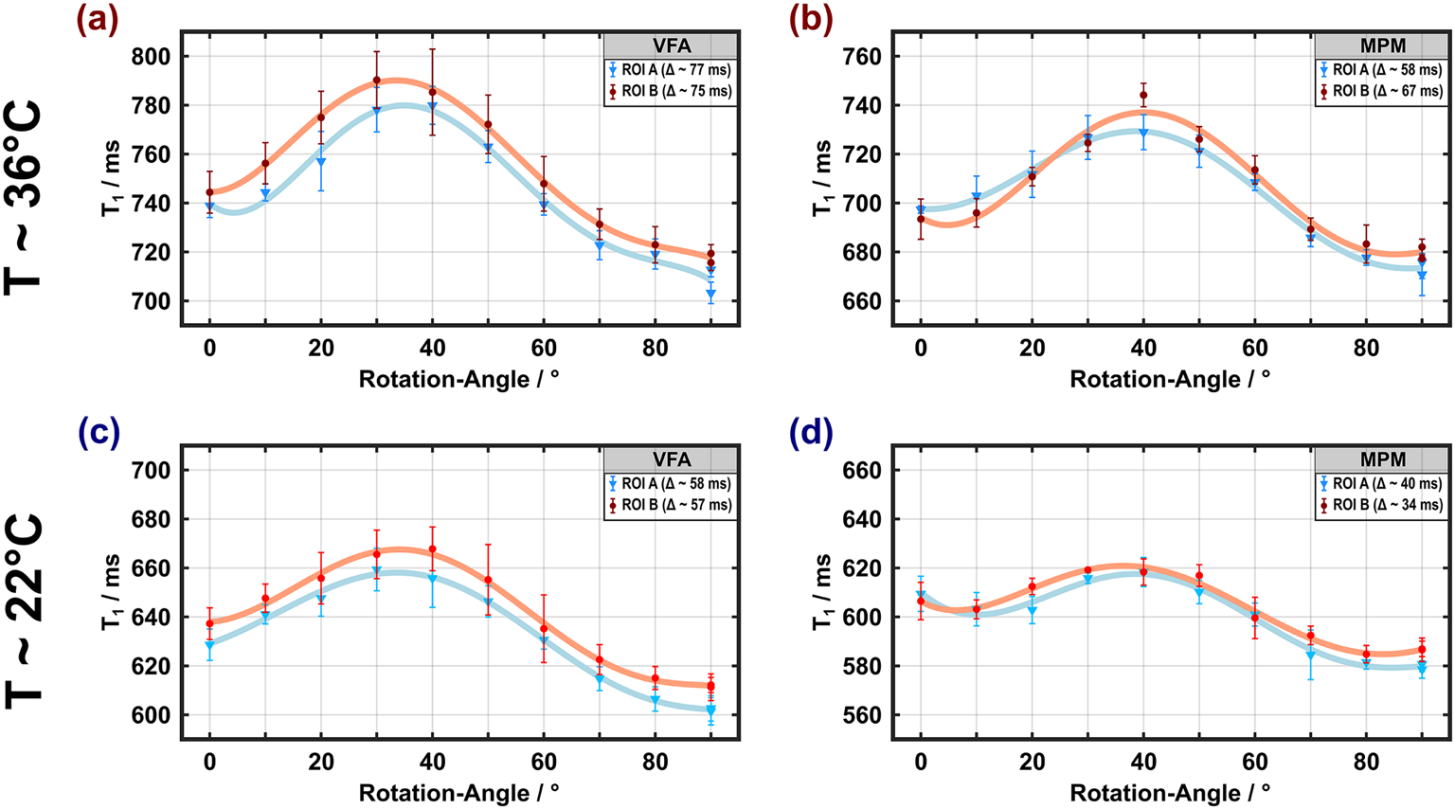
Apparent spin–lattice relaxation times obtained with VFA and MPM at 36 °C (**a**, **b**) and 22 °C (**c**, **d**) as a function of the rotation angle (symbols, lines and ROI definitions as in Fig. 2). The dependence of $T_{1}$ on $\theta_{{{\text{FB}}}}$ is similar to the results obtained with MP2RAGE, however with an increased difference between the maximum and minimum values ($T_{1}^{{{\text{max}}}} - T_{1}^{{{\text{min}}}}$ > 70 ms for VFA and > 55 ms for MPM).

Both VFA and MPM determine $T_{1}$ without inverting the magnetization, in contrast to the IR methods and MP2RAGE. Remarkably, the obtained $T_{1}$ values showed the highest anisotropy (Fig. 5), with variations of about 10% and 8% for VFA ($T_{1}$ = 710–790 ms, Fig. 5a) and MPM ($T_{1}$ = 680–740 ms, Fig. 5b), respectively. Both methods yielded similar trends with maxima at $\theta_{{{\text{FB}}}}$ between 30° and 40°, albeit with reduced $T_{1}$ values for MPM. Compared to the results obtained at 36 °C, the orientation dependence was slightly lower at 22 °C. Unlike IR-based measurements, VFA methods (and, correspondingly, MPM) are susceptible to errors or inhomogeneities of $B_{1}^{ + }$, which limit the accuracy of $T_{1}$ estimations if not properly corrected ^13^. In the current case, our measurements benefitted from a very precise $B_{1}^{ + }$ adjustment, employing a flip-angle array and excellent homogeneity in the central region of the Helmholtz coil (Supplementary Fig. S8). $B_{1}^{ + }$ inhomogeneities across the ROIs were corrected by rescaling the nominal flip angles.

#### MT experiments

**Figure 6:**
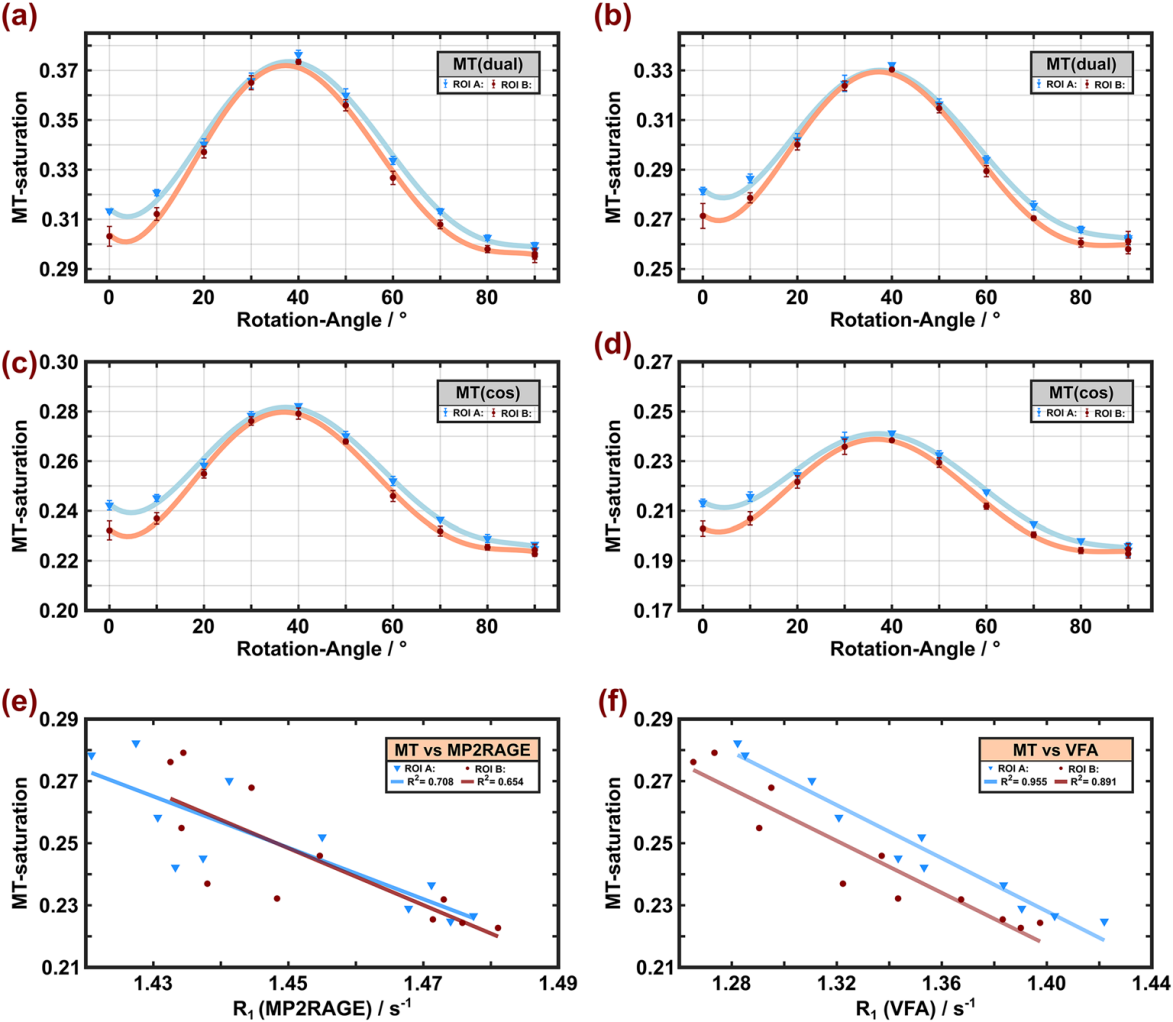
MT-saturation obtained at 36 °C with off-resonant irradiation at an offset frequency of ± 10 kHz with $\gamma B_{{1,{\text{RMS}}}}^{ + } /\left( {2\pi } \right)$ = 500 Hz (**a**, **c**) or 705 Hz (**b**, **d**). Dual-sided saturation was achieved by either alternating the offset-frequency (**a**, **b**) or cosine modulation (**c**, **d**) of the Gaussian MT pulse (symbols, lines and ROI definitions as in Fig. 2). An overall identical orientation dependence with a maximum at $\theta_{{{\text{FB}}}}$ between 30° and 40° was obtained with minor differences in the range of MT_sat_ variation (7–8% and 5–6% for frequency alternation and cosine modulation, respectively. Bottom row, correlations of results from relaxation-rate and MT measurements at 36 °C for both ROIs. Significant correlations were also obtained between $\theta_{{{\text{FB}}}}$-dependent ${\text{MT}}_{{{\text{sat}}}}$ (here exemplarily shown for cos modulation and $\gamma B_{{1,{\text{RMS}}}}^{ + } /\left( {2\pi } \right)$ = 500 Hz) and $R_{1}$ estimates obtained with MP2RAGE (**e**) and VFA (**f)**.

Characteristic $\theta_{{{\text{FB}}}}$-dependent changes in the signal attenuation induced by MT were visible in all MT experiments (Fig. 6a–d, some room temperature results are shown in Supplementary Fig. S10 for comparison). Regardless of the amplitude of the off-resonant MT pulse or the strategy to achieve dual-sided saturation (alternating the offset frequency or cosine modulation), a maximum of ${\text{MT}}_{{{\text{sat}}}}$ was obtained between 30° and 40°. As expected, ${\text{MT}}_{{{\text{sat}}}}$ increased with the higher $B_{1}$ amplitude, whereas the same general orientation dependence was observed in all measurements. Alternating the offset frequency produced a slightly larger range of ${\text{MT}}_{{{\text{sat}}}}$ variation compared to cosine modulation, which may be due to a small amount of dipolar order created during the RF train.

#### Comparison of the results from different relaxometry sequences

The IR and MP2RAGE results showed a high similarity of the $T_{1}$ variation with $\theta_{{{\text{FB}}}}$(Figs. 2, 3 and 4), which was further underlined by high correlations between both methods shown in Fig. 4e, f. However, $T_{1}$ estimates obtained with MP2RAGE were shorter than those obtained with IR, and even more so for IR measurements with only longer TIs (i.e., TIs that better match those used with MP2RAGE). For example, $T_{1}$ values of 735–770 ms were obtained with the adiabatic inversion pulse and TI > 100 ms but only 675–705 ms with MP2RAGE. High correlations were also obtained between ${\text{MT}}_{{{\text{sat}}}}$ and different $T_{1}$ results, demonstrating the impact from the readout pulses in GRE sequences, which lead to MT. Examples are shown in Fig. 6e, f. The GRE approaches (VFA, MPM, MP2RAGE) yielded overall comparable trends of *T*_1_ variation with $\theta_{{{\text{FB}}}}$ but differed considerably in the degree of anisotropy, with up to 10% variation for VFA and only 3–4% for MP2RAGE.

### Analysis of the IR experiments within the BSB model

**Figure 7:**
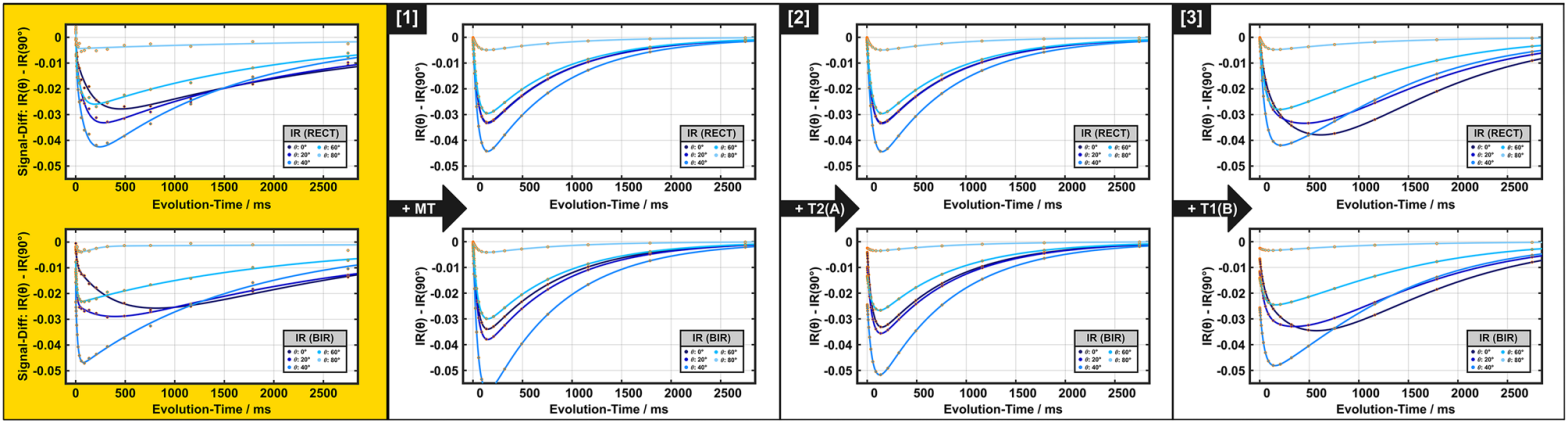
Subset of the IR datasets recorded at different orientation angles (0°, 20°, 40°, 60°, and 80° indicated by different symbols) shown as the difference from the result for $\theta_{{{\text{FB}}}}$ = 90° for both inversion pulses (top row: rectangular hard pulse, bottom row: BIR-4 adiabatic pulse). The solid lines are guides to the eye. Experimental data (left, golden background) is presented as mean over ROI A. For comparison, BSB model forward simulations with different levels of combinations of anisotropic parameters are shown: *(1)* Assumption of orientation-dependent partial inversion/saturation of pool *B* by RF application; *(2)* assumption of orientation-dependence of the transverse relaxation time $T_{2}^{A}$ in addition to the first model; *(3)* assumption of orientation dependence of the longitudinal relaxation time $T_{1}^{B}$ in addition to the second model. Note that crossings of the transients of different rotation angles observed experimentally are only observed with model *(3)*.

**Figure 8:**
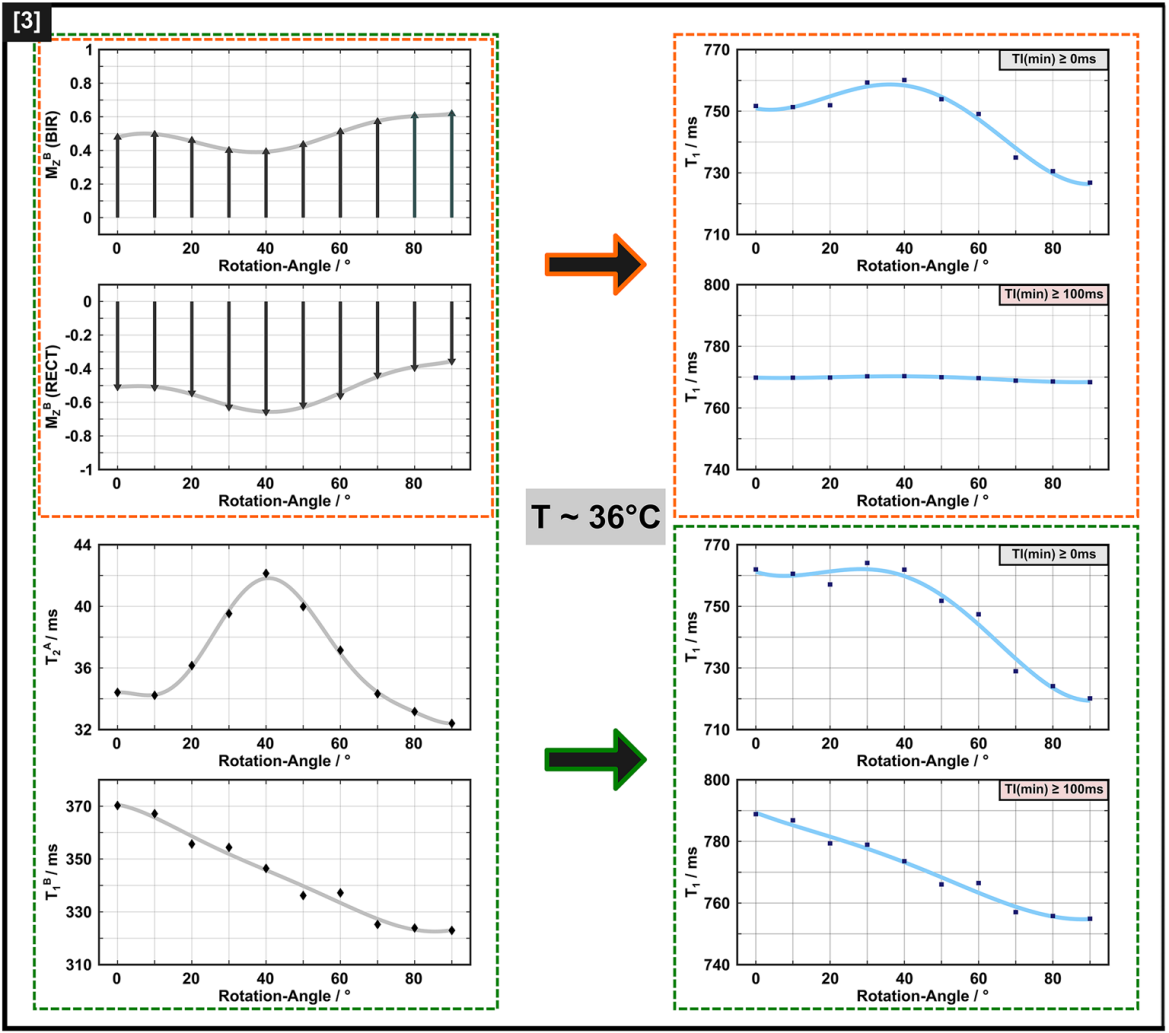
Overview of anisotropic BSB model parameters as a function of $\theta_{{{\text{FB}}}}$ (left column), obtained from the analyses illustrated in this figure and resulting orientation-dependent $T_{1}$ (right column) from fits to the IR data. The additional $\theta_{{{\text{FB}}}}$-independent BSB parameters were MPF = 0.17, $T_{1}^{A}$ = 1002 ms, and $k$ = 16.9/s. Solid lines are guides to the eye. The illustrations in frames with broken orange lines show the orientation dependence of $M_{z}^{B} \left( {0^{ + } } \right)$ according to model *(1)*, with partial saturation obtained with the 5 ms BIR-4 pulse and partial inversion obtained with the 40 µs hard pulse. To better visualize the differences in $M_{z}^{B} \left( {0^{ + } } \right)$, grey arrows indicate that $M_{z}^{B} \left( {0^{ + } } \right)$ after the BIR-4 pulse still points along the external magnetic field, whereas it is partially inverted after application of the RECT pulse. This model yields orientation-dependent $T_{1}$ if all TIs are included in the monoexponential fitting. However, it leads to almost invariant $T_{1}$ when restricting the analysis to TI > 100 ms, which is inconsistent with the experimental data in Fig. 2. The trends in the anisotropy of $T_{1}$ estimates obtained both with all TIs or only those > 100 ms could be reproduced by assuming additional orientation dependencies of $T_{2}^{A}$ and $T_{1}^{B}$ (illustrations in frames with the broken green lines). Consistent trends were also obtained when only TIs > 450 ms were considered (figures not shown).

Results from ROI A obtained at 36 °C from BSB model analyses with different considerations of orientation dependence are shown in Figs. 7 and 8 (results for data acquired at 22 °C are shown in Supplementary Fig. S11). Since ${\text{MT}}_{{{\text{sat}}}}$ changed significantly with $\theta_{{{\text{FB}}}}$ (Fig. 6a–d), $M_{z}^{B} \left( {0^{ + } } \right)$ was assumed to be orientation dependent in model *(1)* due to a variable efficiency of the inversion pulse. Taking into account the differences in $M_{z}^{B} \left( {0^{ + } } \right)$, as shown in Fig. 8 (dashed orange box), the associated effect of the build-up of a maximum signal difference could be reproduced qualitatively [Fig. 7, model *(1)*]. However, if orientation effects would impact only $M_{z}^{B} \left( {0^{ + } } \right),$ both pools would relax in synchrony once the populations are equilibrated. Therefore, model *(1)* fails to explain the observed crossings of transients that were obtained for different $\theta_{{{\text{FB}}}}$ (Fig. 7, left column). This is further evident in Fig. 8 (dashed orange box): Model *(1)* yielded orientation dependence of $T_{1}$ if all TIs were included in the monoexponential fitting but failed to explain the experimental result for analyses including only TI > 100 ms. Model *(2)* achieved some refinement of the fits suggesting an orientation-dependent variation of $T_{2}^{A}$ between 34 and 42 ms (Fig. 8). Like model *(1)*, however, it did not explain the dynamic behavior of transients as a function of TI (Fig. 7).

Finally, spin–lattice relaxation in the semi-solid pool was assumed to be anisotropic in model *(3)*. This therefore considered MPF fixed at 0.17 (obtained from the first-level fitting), $k$ and $T_{1}^{A}$ as free isotropic BSB parameters, $T_{2}^{A} \left( {\theta_{{{\text{FB}}}} } \right)$ and $T_{1}^{B} \left( {\theta_{{{\text{FB}}}} } \right){ }$ as free orientation-dependent parameters, as well as $M_{{z,{\text{RECT}}}}^{B} \left( {0^{ + } ,\theta_{{{\text{FB}}}} } \right)$ and $M_{{z,{\text{BIR - 4}}}}^{B} \left( {0^{ + } ,\theta_{{{\text{FB}}}} } \right)$ as free orientation-dependent parameters that were allowed to differ for the different types of inversion pulses (rectangular and BIR-4, respectively). Figure 8 shows that all trends in the anisotropy of $T_{1}$ estimates were reproduced with this model, including a subtle peak at $\theta_{{{\text{FB}}}}$ = 30–40° for experiments with a hard-pulse and a monotonic $T_{1}$ decay upon increasing $\theta_{{{\text{FB}}}}$ from 0° to 90°.

## Discussion

In this study, we demonstrate a reproducible orientation dependence of the apparent $T_{1}$ in fixed porcine spinal cord WM. We avoided potential confounds due to differences in myelination, present in previous work comparing different WM regions, by direct sample reorientation. Furthermore, by combining different techniques to measure $T_{1}$, the hypothesis that inconsistencies in the observed orientation dependence of $T_{1}$ might be related to the acquisition method could be directly addressed and confirmed. Overall, the results of both selected ROIs were consistent in terms of the orientation dependence. The differences can presumably be attributed to small changes in the degree of myelination along the spinal cord.

### Anisotropy of ${\varvec{T}}_{1}$

All measurements showed some degree of $T_{1}$ dependence on $\theta_{{{\text{FB}}}}$. However, the degree of such anisotropy ranged from approximately 10% obtained with a standard MPM protocol to minor changes of 2–3% for IR experiments with TI > 100 ms. The overall trend of the orientation dependence (an almost monotonic $T_{1}$ decrease with increasing $\theta_{{{\text{FB}}}}$ vs. occurrence of a maximum at $\theta_{{{\text{FB}}}}$≈30–40°) demonstrated further variability for different protocols or different ranges of acquisition parameters, as shown for the mono-exponential fitting of distinct subsets of the IR data. It is elusive to gain a better understanding of the mechanism underlying orientation dependence from a simplified analysis of the IR curves, especially when considering the differences in remaining residuals and also the weighting of the rapidly relaxing component (visible as a strong deviation for short TIs, Supplementary Fig. S9). However, the apparent orientation effect always contains a meaningful (‘true’) biological contribution, even if bias is present due to a restricted range of experimental data (e.g., no acquisition at very short TI) or an oversimplified model (e.g., limited number of proton pools). This is supported by the fact that the trends of $T_{1}$ variation, as a function of $\theta_{{{\text{FB}}}}$, could be reproduced with the BSB model for different subsets of the data (Fig. 8).

The correlation between the simple, model-free parameter $${\text{MT}}_{{{\text{sat}}}}$$ and $$R_{1} = 1/T_{1}$$ (Fig. 6e, f) suggests that MT anisotropy shines through in the $$T_{1}$$ measurements to a variable extent, depending on the specific relaxometry method. Anisotropy of MT in cerebral WM, due to cross-relaxation between water protons and membrane lipids, is well established and related to the cylindrical arrangement of myelin enveloping axons ^25^. The peak of $$T_{1}$$ around 30°–40° is therefore explained by the specific arrangement of lipids in the myelin sheath, leading to an orientation-dependent lineshape for the myelin proton pool as discussed by Pampel et al. ^25^ As shown in Fig. 2 of Ref. ^25^, there is a remarkable change in the lineshape and amplitude in the angle range between 30° and 40°. When applying RF pulses of limited bandwidth, the degree of saturation will, therefore, undergo a marked orientation-dependent change as a function of the fiber orientation in this range that is transferred via cross-relaxation to the water protons and, thereby, affects the apparent $$T_{1}$$. Remarkably high correlations were observed in the VFA experiments ($$R^{2}$$ = 0.89–0.96, Fig. 6f), which employ a large number of (readout) RF pulses. This agrees with previous work pointing to the sensitivity of VFA experiments to MT effects ^12^. We note that the VFA and MPM acquisitions were not compensated for MT effects in our experiments, as recently proposed using multiband RF pulses ^14^. Thus, sensitivity to MT effects is expected, which changes with orientation, as established by the observed correlations. A comparison between the VFA and MPM results is also complicated due to distinct TRs and flip angles, which influence the *T*_1_ estimates. Further experimental evidence that MT anisotropy shines through in these measurements arises from the comparison of the temperatures. In analogy to the reduced orientation dependence of MT saturation at room temperature (Fig. 6a–d and Supplementary Fig. S10), the degree of anisotropy for $$T_{1}$$ obtained with VFA and MPM was also reduced. For MP2RAGE, this correlation was lower ($$R^{2}$$ = 0.65–0.71$$)$$, but the general trend of an increased $$R_{1}$$ at orientations of most effective MT saturation was identical (Fig. 6e). Note that the flip angles in the MP2RAGE readout were smaller than in VFA acquisitions. The trend toward consistently shorter WM $$T_{1}$$ values obtained with MP2RAGE is in line with *in-vivo* results ^53,54^.

The observed differences at the two temperatures are not limited to an overall longer $T_{1}$ at 36 °C compared to 22 °C, but also affect the $T_{1}$-dependence on $\theta_{{{\text{FB}}}}$. This is particularly evident for the IR (Figs. 2a, d and 3a, d) and MP2RAGE results (Fig. 4a–d). Differences in the spin–lattice relaxation behavior of ex vivo WM tissue at room temperature and body temperature have already been observed in previous work and attributed to a change in the cross-relaxation dynamics ^3^. Changes of the microstructure and water mobility due to tissue fixation have also been reported, which may contribute to differences in the relaxation dynamics ^55^. This suggests that not all factors of *T*_1_ contrast are preserved *post mortem*, which may affect a method-dependent degree of observed anisotropy. Thus, a quantitative translation to in vivo observations is not straightforward. Nevertheless, the studied anisotropy effect is mainly caused be ordered fiber arrangement (as indicated by a high FA). Recent research also indicated that fixation preserves the degree of anisotropy compared to in vivo results in mouse brain ^56^.

### Translating orientation dependence into biophysical parameters

To obtain an explanation for $T_{1}$ anisotropy in terms of physical parameters, special attention was paid to the IR measurements, which provide the most extensive TI range. They also confirm previous observations that the adiabatic condition was not completely fulfilled with the BIR-4 pulse due to high dipolar coupling strength ^13^. Within the limits of the BSB model, this results in unequal magnetization states of the free and semi-solid proton pools and, consequently, to biexponential relaxation ^57^. As a consequence, monoexponential fitting results in an apparent $T_{1}$ that is shorter compared to the result after a full inversion producing equal initial spin states ^13^. In comparison, the short rectangular pulse leads to a more complete inversion of the non-aqueous protons, and the recovery is better characterized by monoexponential behavior resulting in a longer apparent $T_{1}$ (Figs. 2a and 3a and Supplementary Fig. S9).

The influence of MT processes in biological tissues is well understood in two-pool systems ^58^. Although more sophisticated models for WM have been introduced ^2,3,59^, they require too many parameters to be reliably assessed with our measurements. Therefore, we restrict our discussion to the simplified BSB model, which explains our central findings.

Differences in $M_{z}^{B} \left( {0^{ + } } \right)$ lead to different deviations from their equilibrium states of the two spin pools, provided that $M_{z}^{A} \left( {0^{ + } } \right)$ is almost perfectly inverted. This in turn has an effect on the recovery curve and thus on the $T_{1}$ estimate obtained with monoexponential fitting. The assumption of orientation dependence of $M_{z}^{B} \left( {0^{ + } } \right)$ is in line with earlier observations of orientation dependence of $T_{2}^{B}$, which was attributed to the cylindrical symmetry of the myelin sheath around axons ^25^. Notably, the obtained trend for $M_{z}^{B} \left( {0^{ + } } \right)$, which is minimal (i.e., most efficient inversion produced by the hard pulse or strongest partial saturation produced by the BIR-4 pulse) at $\theta_{{{\text{FB}}}}$ around 30°–40° (Fig. 8) agrees well with the angle range of the longest apparent $T_{2}^{B}$ values observed by Pampel et al. ^25^. The time required for magnetization exchange between non-aqueous and aqueous protons is in the order of 100 ms according to the literature ^13,59^. This correlates well with the range of TIs, for which we observed the largest signal differences (4–5%) for different $\theta_{{{\text{FB}}}}$ (Fig. 7).

$M_{z}^{B} \left( {0^{ + } } \right)$ captures effects characterizing the semi-solid proton pool, which is not readily visible to MRI. For $M_{z}^{A}$ inversion, the carefully adjusted 40 µs hard pulse achieved largely ideal conditions. However, this was not the case for the adiabatic pulse ($\tau_{p}$ = 5 ms) and different efficiencies may result from anisotropy of $T_{2}^{A}$. Orientation dependence of $T_{2}$ in cerebral WM was already reported by others ^60,61^, albeit with a smaller effect compared to our results shown in Fig. 8. Anisotropy of $T_{2}^{A}$ in the BSB model may be attributed to diffusion of water through magnetic field inhomogeneities near myelinated axons ^62^, which would be expected to be more pronounced with increased diffusivity ^63^. This assumption is supported by the fitted BSB model parameters of the room-temperature measurements (Supplementary Fig. S11), which showed a reduced degree of $T_{2}^{A}$ orientation dependence. Generally, *transverse* relaxation effects (i.e., $T_{2}^{A}$ or $T_{2}^{B}$) may impact $M_{z} \left( {0^{ + } } \right)$ via $M_{z}^{A} \left( {0^{ + } } \right)$ or $M_{z}^{B} \left( {0^{ + } } \right)$, due to deviation from the adiabatic condition or relaxation during the inversion pulse. However, they do not cause transient effects during the recovery of *longitudinal* magnetization, as observed in our IR experiments, which suggests orientation-dependent longitudinal relaxation. Considering that orientation dependence has been firmly demonstrated for $T_{2}^{B}$ characterizing dipolar line broadening for the semi-solid pool ^25^, it is only logical to assume that the same residual dipolar couplings lead to anisotropic $T_{1}^{B}$. By contrast, the intrinsic $T_{1}^{A}$ of the highly mobile water pool should be isotropic. Remarkably, the decay of $T_{1}^{B}$ with increasing $\theta_{{{\text{FB}}}}$ (Fig. 8) obtained with the BSB analysis and model *(3)* is in excellent agreement with recent theoretical predictions from a lateral diffusion model for bound protons in axon bundles ^4^. This model can explain the differences in longitudinal relaxation for longer TIs, or equivalently, after equilibration of both pools (Figs. 7 and 8). Despite this agreement, we cannot entirely exclude orientation dependence for other BSB parameters, such as the exchange rate $k$, which may result, for example, from an anisotropic nuclear Overhauser effect (NOE). A reliable clarification of this possibility, however, would require additional measurements, which are beyond the scope of our study. Of note, our estimate of $T_{1}^{B}$, varying between 320 and 370 ms with $\theta_{{{\text{FB}}}}$ (Fig. 8), is in reasonable agreement with the range of recent estimates in fresh or fixed tissue ^2,6^.

### Anisotropy of ${\varvec{T}}_{1}$—pitfall or benefit?

In general, orientation-related variation of the apparent $T_{1}$, although it may contribute up to 10%, is not the dominant contributor to observed differences in $T_{1}$ in WM, which may range from 690 to 1100 ms in different brain regions at 3 T ^11^. A common approach in quantitative MR studies is to correlate the measured $T_{1}$ (or $R_{1}$) of tissues with the macromolecular mass fraction or with the water content $f_{w}$ ^64^. A simple attempt to explain tissue $T_{1}$ is the solvation-layer model, which predicts $R_{1} = f_{w}^{ - 1} + {\text{const}}$ in the limit of fast exchange ^65^. A strong correlation of $R_{1}$ with the tissue’s water content is often reported, including WM ^8^. A 10% uncertainty in the measured $T_{1}$ due to orientation effects (the maximal effect observed here depending on the relaxometry method) would, therefore, correspond to an equally large uncertainty in the estimated water content. A similar consideration applies to the macromolecular content.

On the other hand, anisotropic relaxation processes, which may include longitudinal or transverse relaxation or also MT (i.e., cross-relaxation) effects, provides a benchmark for the validation of biophysical models. For example, a distinct orientation dependence offers a link to specific components of the microstructure, such as myelinated axons in WM with approximately cylindrical symmetry. Examples include predictions based on the cylindrical lineshape model for MT saturation ^25^ or spin–lattice relaxation of bound protons within the lateral diffusion model ^4^, both of which are in line with our observations.

## Conclusion

Longitudinal proton relaxation in WM depends on orientation, which is attributed to microstructural anisotropy due to the presence of bundles of approximately cylindrical nerve fibers (myelinated axons). The arrangement of associated membrane components is sensed by mobile water protons through dipolar cross-relaxation and/or chemical exchange. The extent of observed orientation-dependent $T_{1}$ variation depends on the exact experimental conditions and, thus, on the relaxometry pulse sequence, in particular, on the type, amplitude and number of applied RF pulses. A sufficient description of the observed effects is already obtained with the BSB model for MT, which considers only two microanatomical compartments (mobile water and semi-solid “macromolecules”). The results suggest that $T_{2}^{B}$, which characterizes the dipolar lineshape, and also $T_{1}^{B}$ of the semi-solid pool are anisotropic. This corroborates previous interpretations of MT anisotropy. Remarkably, the results also suggest anisotropy of $T_{2}^{A}$ of the mobile pool. In summary, our findings clarify apparent inconsistencies in the literature and demonstrate that orientation-dependent $T_{1}$ provides a valuable link to microstructure beyond simple correlation analyses.

### Supplementary Information

Supplementary Information.

## Data Availability

Pre-processed MR data and Matlab scripts will be made available upon acceptance at https://dataverse.harvard.edu/ (10.7910/DVN/EUAVIX). Binary code of the pulse sequences used in the current work can be made available upon written request to the authors for use at other institutions on compatible scanners. Note that pulse sequences implemented with IDEA (Integrated Development Environment for Applications; Siemens Healthineers) contains vendor-specific code. Therefore, sharing sequence code can only take place via the customer-to-customer partnership program (so-called C2P procedure).
