# Supplementary material for: Anisotropic longitudinal water proton relaxation in white matter investigated ex vivo in porcine spinal cord with sample rotation
Source: Sci Rep. 2024 Jun 5;14:12961. doi: 10.1038/s41598-024-63483-0 (PMC11153615; doi:10.1038/s41598-024-63483-0)
Supplement: Supplementary file 1 — Supplementary Information. [file 41598_2024_63483_MOESM1_ESM.pdf]

Supplementary Information

Anisotropic Longitudinal Water Proton Relaxation Investigated *Ex Vivo* in Porcine Spinal Cord White Matter with Sample Rotation

Niklas Wallstein[1], André Pampel[1], Carsten Jäger[2,3], Roland Müller[1], and Harald E. Möller[1,4]

[1] NMR Methods & Development Group, Max Planck Institute for Human Cognitive and Brain Sciences, Leipzig, Germany

[2] Department of Neurophysics, Max Planck Institute for Human Cognitive and Brain Sciences, Leipzig, Germany

[3] Paul Flechsig Institute – Center of Neuropathology and Brain Research, Medical Faculty, Leipzig University, Germany

[4] Felix Bloch Institute for Solid State Physics, Leipzig University, Germany

Supplementary Methods

Binary Spin-Bath Model and Homogeneous Bloch-McConnell Equations

As discussed in detail in previous work (1), we use simplified Bloch-McConnell equations (2,3) to describe the evolution of the total magnetization in a water proton pool A and a semi-solid non-aqueous proton pool B, and rewrite them in a homogeneous form according to

$$\dot{\mathbf{M}} = -\mathbf{L} \cdot \mathbf{M}, \tag{S1}$$

with

$$\mathbf{M} = \begin{pmatrix} 1/2 & M_x^A & M_y^A & M_z^A & M_z^B \end{pmatrix}^T, \tag{S2}$$

and the dynamic matrix

$$\mathbf{L} = \begin{pmatrix} 0 & 0 & 0 & 0 & 0 \\ 0 & R_2^A & \Omega & -\omega_{1y} & 0 \\ 0 & -\Omega & R_2^A & \omega_{1x} & 0 \\ -2R_1^A M_0^A & \omega_{1y} & -\omega_{1x} & R_1^A + kM_0^B & -kM_0^A \\ -2R_1^B M_0^B & 0 & 0 & -kM_0^B & R_1^B + kM_0^A + R_{\mathrm{RF}}^B(\omega_1, \Omega, T_2^B) \end{pmatrix}. \tag{S3}$$

M_x, M_y and M_z are the cartesian magnetization components in the rotating frame, M_0 is the equilibrium magnetization, $R_1 = 1/T_1$ and $R_2 = 1/T_2$ are the longitudinal and transverse relaxation rates, respectively, and the superscripts A and B denote the respective proton pool. The MT rate constant is denoted as k, which is different from the notation used by Müller et al. (1), but agrees with Manning et al. (4). The radiofrequency (RF) field is applied with a frequency ω_{rf} and amplitude $B_1 = -\omega_1/\gamma$ with transverse components B_{1x} and B_{1y}. $\Omega = \omega_0 - \omega_{\mathrm{rf}}$ is the offset frequency, ω_0 the Larmor frequency,

and γ the gyromagnetic ratio of the proton. Transverse magnetization of the semi-solid pool B and, hence, transverse magnetization transfer (MT), is neglected due to its rapid decay (5). The function $R_{\mathrm{RF}}^{B}(\omega_1, \Omega, T_2^B)$ in the matrix element $L_{5,5}$ accounts for the effect of RF pulses on M_z^B. For MT experiments, it is typically modeled as a saturation rate (2,6),

$$R_{\mathrm{RF}}^{B}(\omega_1, \Omega, T_2^B) = \pi\omega_1^2 g^B(\Omega, T_2^B), \tag{S4}$$

with an appropriate absorption lineshape function $g^B(\Omega, T_2^B)$ of the semi-solid pool. In particular, a super-Lorentzian (7) or a modified super-Lorentzian accounting for the cylindrical symmetry of myelinated axons (8) have been employed for cerebral white matter (WM). The assumption of a (partial) saturation of the semi-solid pool at a rate R_{RF}^{B} is useful for typical MT experiments on clinical scanners with long pulse durations (order of milliseconds) and relatively weak RF irradiation but becomes invalid for strong pulses with $\gamma^2 B_1^2 T_1^B T_2^B \gg 1$.

To solve Eq. S1, it is convenient to divide the pulse sequence into individual periods Δt_i (i = 1, 2, ..., n) for which $\mathbf{L}$ is constant, which is generally fulfilled for delays between RF pulses or incremental time steps of the digitized pulses (1,9). This allows to define propagators

$$\mathbf{P}^{(i)} = \exp\left(-\Delta t_i \mathbf{L}^{(i)}\right) \tag{S5}$$

given by the matrix exponential of $\Delta t_i \mathbf{L}^{(i)}$, and the evolution of the magnetization can be calculated with arbitrary precision as

$$\mathbf{M}\left(t + \sum_{i=1}^{n} \Delta t_i\right) = \left(\prod_{i=0}^{n-1} \mathbf{P}^{(n-i)}\right) \cdot \mathbf{M}(t) \tag{S6}$$

employing numerical methods (10).

Supplementary Tables

Supplementary Table S1. Acquisition parameters used for IR, MP2RAGE, VFA and MPM 1D projections without slice selection and phase encoding.

Parameter	IR	MP2RAGE	VFA	MPM
**TR / ms**	13000	1850	—	—
**GRE TR / ms**	-	4.0	30.0	18.0
**Readout pulse**	RECT	RECT	RECT	RECT
α **/ °**	90	8 (4)	4, 8, 12, 16, 20, 24, 28, 32, 40, 50, 60	4 / 25
τ_p **/ µs (readout)**	20	100	100	100
**Inversion pulse**	RECT / BIR-4	BIR-4	—	—
τ_p **/ µs (inversion)**	40 / 5000	5000	—	—
**k-space samples**	—	72	—	—
**k-space center**	—	25	—	—
N_{rep}	—	20	196	196
**TI / ms**	0.77, …, 10000	[300, 900], [200, 1200], [600, 1500], [130, 1510], [180, 1400], [230, 1300], [280, 1210], [330, 1100], [380, 1000], [430, 910], [480, 850], [310, 700], [600, 1490]	—	—
**Total Acquisition-Time / min:s**	04:59	08:01	2:45	0:30

Supplementary Table S2. Mean spin-lattice relaxation times T_1 (average of the values of all pixels) within the region of interest (ROI; here, the entire sample) and standard deviations (SDs; variation over the ROI) obtained at room temperature in individual measurements in the water phantom. Considering small differences related to temperature fluctuations, all methods show an excellent agreement. Note that the (uncorrected) SDs for the VFA and MPM experiments (marked with an asterisk) exceed those for the IR and MP2RAGE measurements by an order of magnitude. They improve after correction for B_1^+ inhomogeneity.

**Method**	**Temp. / °C**	**Mean T_1 / ms**	**SD / ms**
Inversion recovery			
RECT (τ_p = 40 µs)	20.8	747.4	5.6
RECT (τ_p = 40 µs)	21.4	756.6	5.5
RECT (τ_p = 40 µs)	21.5	757.7	5.2
RECT (τ_p = 40 µs)	21.6	762.0	4.9
BIR-4 (τ_p = 5 ms)	20.9	749.2	5.1
MP2RAGE			
α = 4° (1st)	20.9	751.0	6.8
α = 4° (2nd)	21.5	763.5	7.4
α = 8°	21.0	752.7	14.8
VFA			
α = [4°, …, 60°]	21.4	756.5	77.3*
dto after B_1^+ correction	21.4	752.6	27.1
MPM			
α = [4°, 25°]	21.4	754.8	77.4*
dto after B_1^+ correction	21.4	750.9	25.1

Supplementary Table S3. Average residuals for monoexponential fitting of normalized IR experiments considering either all data points or only those with TI>100 ms or TI>450 ms. Generally, the recovery is better approximated by monoexponential behavior for longer TI, as indicated by smaller average residuals.

**Inversion pulse**	**Temperature**	**Residuals / %**		
		TI > 0 ms	TI > 100 ms	TI > 450 ms
**RECT**	36 °C	4.3	2.8	1.7
	22 °C	7.1	3.8	2.0
**BIR-4**	36 °C	17.3	3.0	1.5
	22 °C	19.0	3.5	1.8

Supplementary Table S4. Confidence intervals of apparent T_1 obtained with fits of the data obtained with different methods to Eqs. 2–5.

**Method**	**Confidence interval / ms**	
	36 °C	22 °C
**IR (Rect)**		
TI > 0 ms	13.5	20.5
TI > 100 ms	23.6	31.5
TI > 450 ms	38.5	48.1
**IR (BIR-4)**		
TI > 0 ms	52.1	55.2
TI > 100 ms	29.5	35.2
TI > 450 ms	40.0	49.4
**MP2RAGE**		
13 TI pairs	13.3	12.2
3 TI pairs	51.1	51.0
**VFA**		
	44.3	25.4

Supplementary Figures

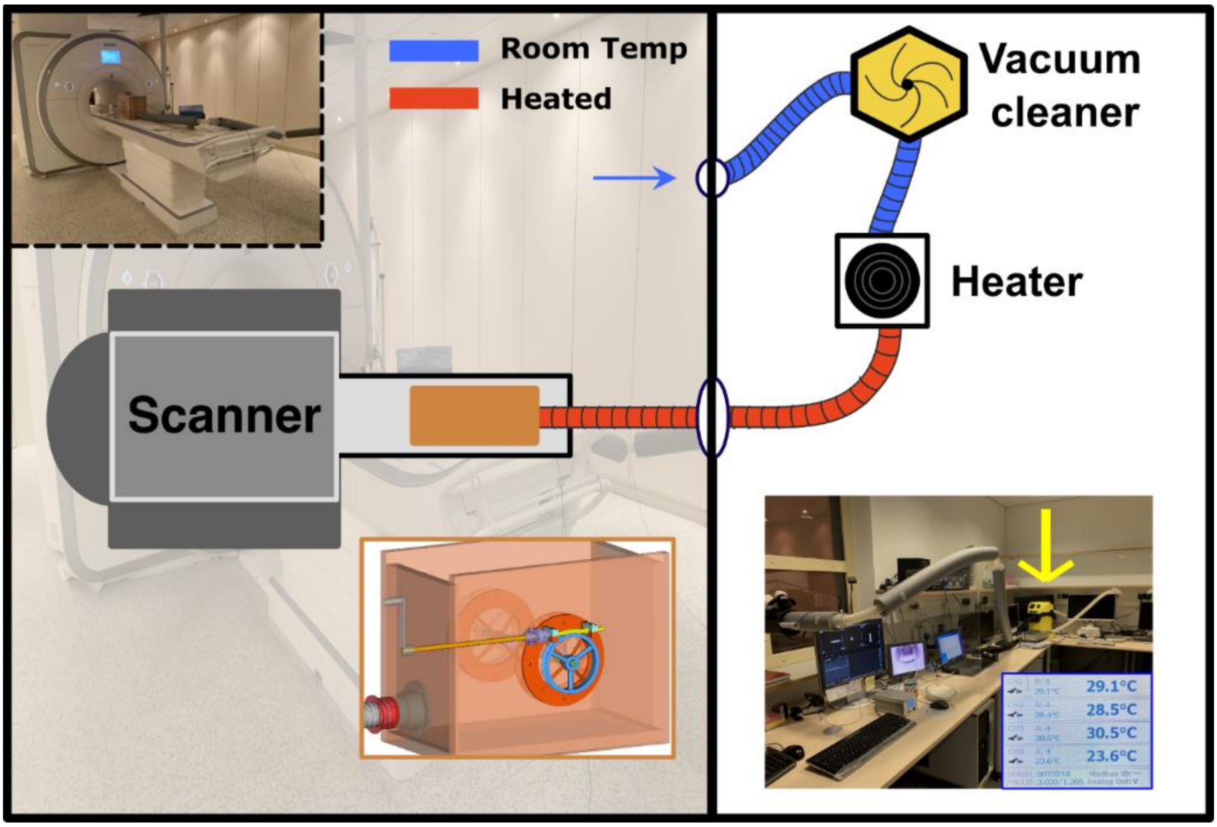

Supplementary Figure S1. Illustration of the setup supporting experiments at elevated temperature by directing a heated airflow from a regulable heater through a wooden box containing the RF coil and sample (see also Figure 1). The box is closed by a wooden lid (not shown) after installing the sample. A universal joint combined with a worm gear on the outside of the permits to rotate the coil and sample via a wooden crank without removing the setup from the magnet's isocenter.

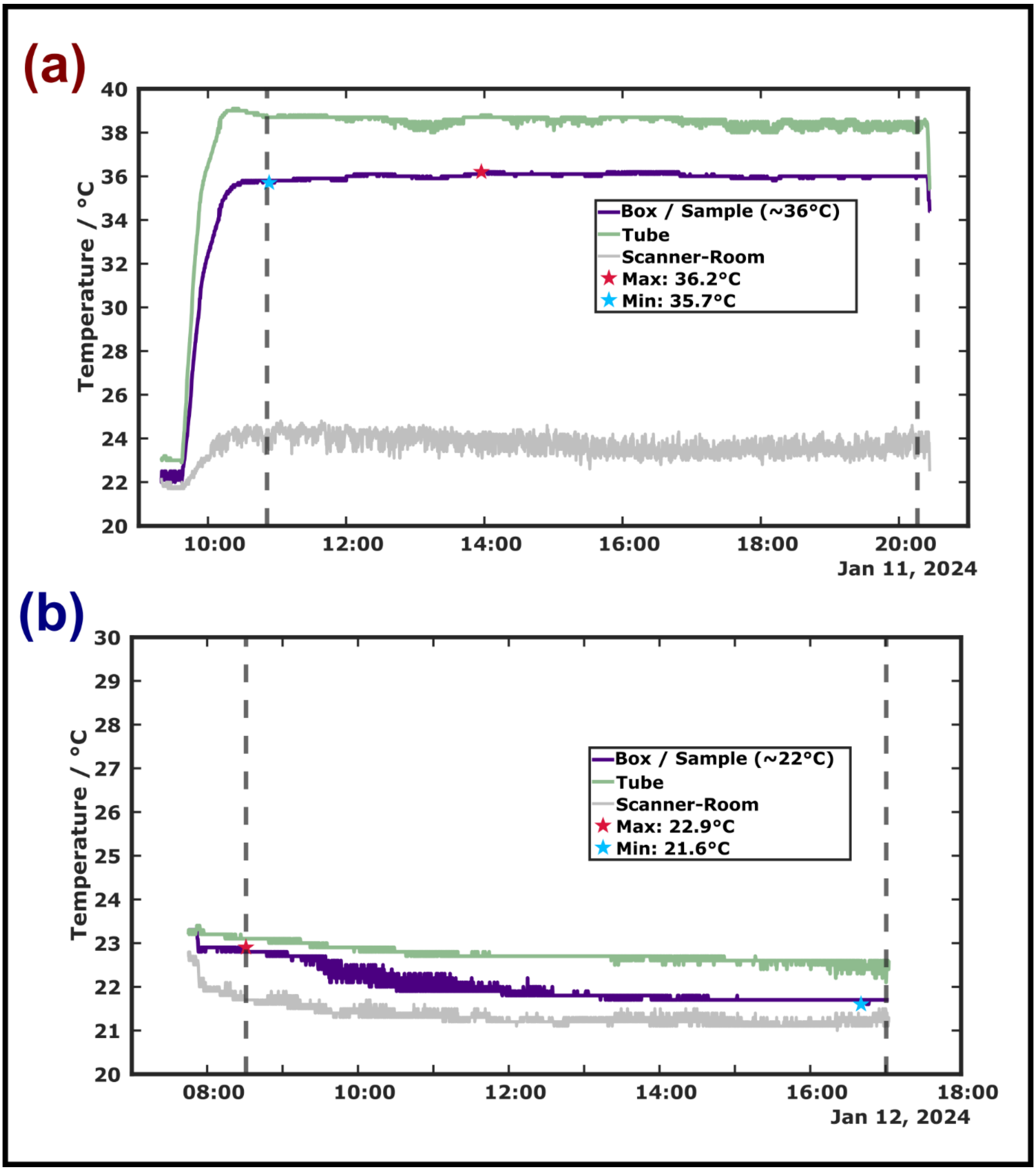

Supplementary Figure S2. Monitored temperatures during two scanning sessions using the fiberoptic sensors at three different positions: inside the box near the sample (violet), inside the tubing for directing the heated airflow into the box (green), and inside the scanner room (gray). **(a)** experiments at approximately body temperature (≈36 °C); **(b)** experiments at room temperature (≈22 °C). Note the excellent stability at elevated temperature achieved with the simple temperature control unit.

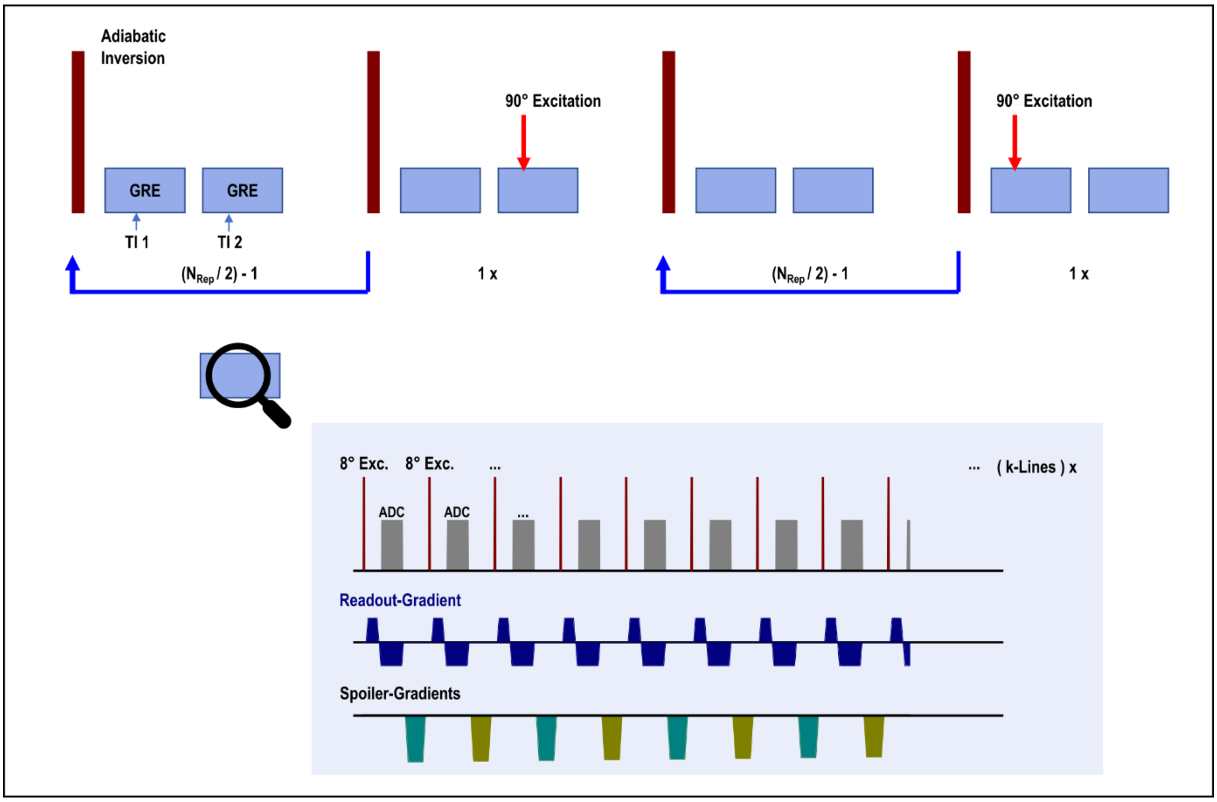

Supplementary Figure S3. Sequence diagram of the modified MP2RAGE sequence for 1D imaging. The number of repetitions, N_{rep}, corresponds to the number of inversion pulses, and hence, steps through the second phase-encoding direction (outer loop) in standard 3D MP2RAGE. Blue boxes labeled "GRE" indicate the two gradient-echo blocks (inner loop), which are shown at more detail at the bottom. Note that slice-selection and phase-encoding gradients are omitted in the GRE kernel. Different colors for the spoiler gradients represent two orthogonal axes along which the gradients are applied in alternating order and with decreasing amplitude. The 90° readout pulse may be shifted away from the center of the GRE-block for partial-Fourier acquisitions as in the current case. In practice, all ADC events of a regular imaging MP2RAGE sequence were present (to confirm that a steady state was reached). For the analysis and fitting, then only one measured signal was used.

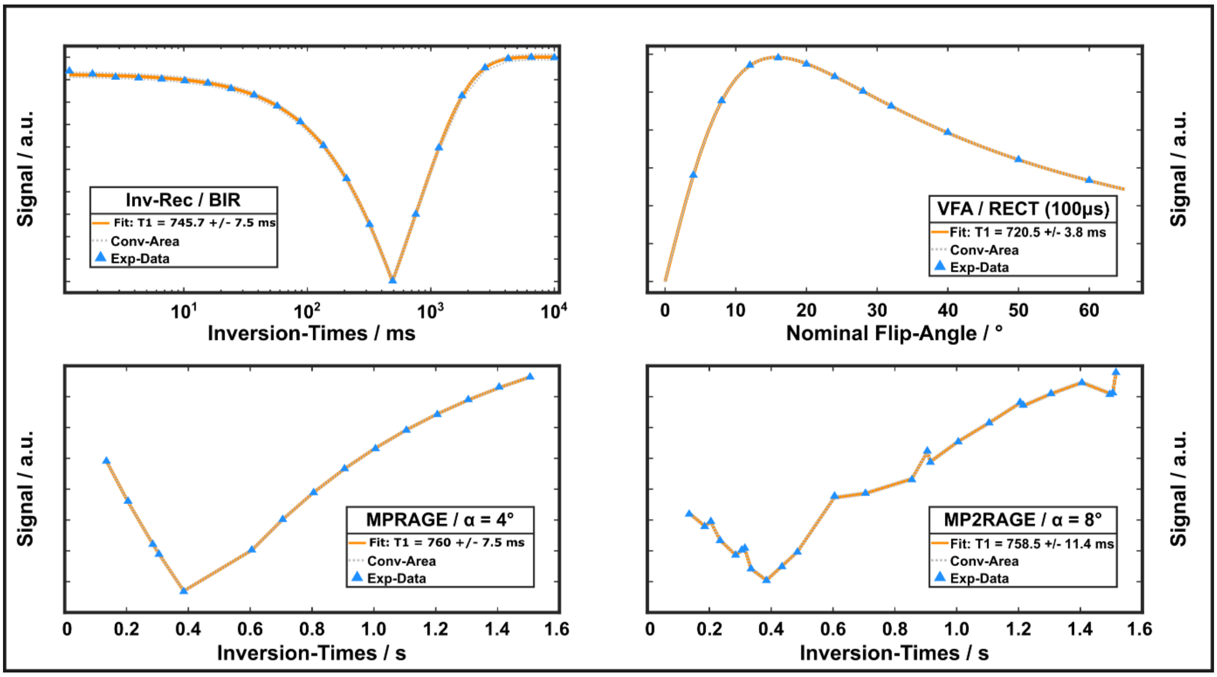

Supplementary Figure S4. Example results obtained in the doped water in arbitrarily selected individual pixels within the region of interest (ROI). Experimental data (magnitude signals) and the fits are shown as blue triangles and orange solid lines, respectively. Estimated confidence intervals are ≤1.5%.

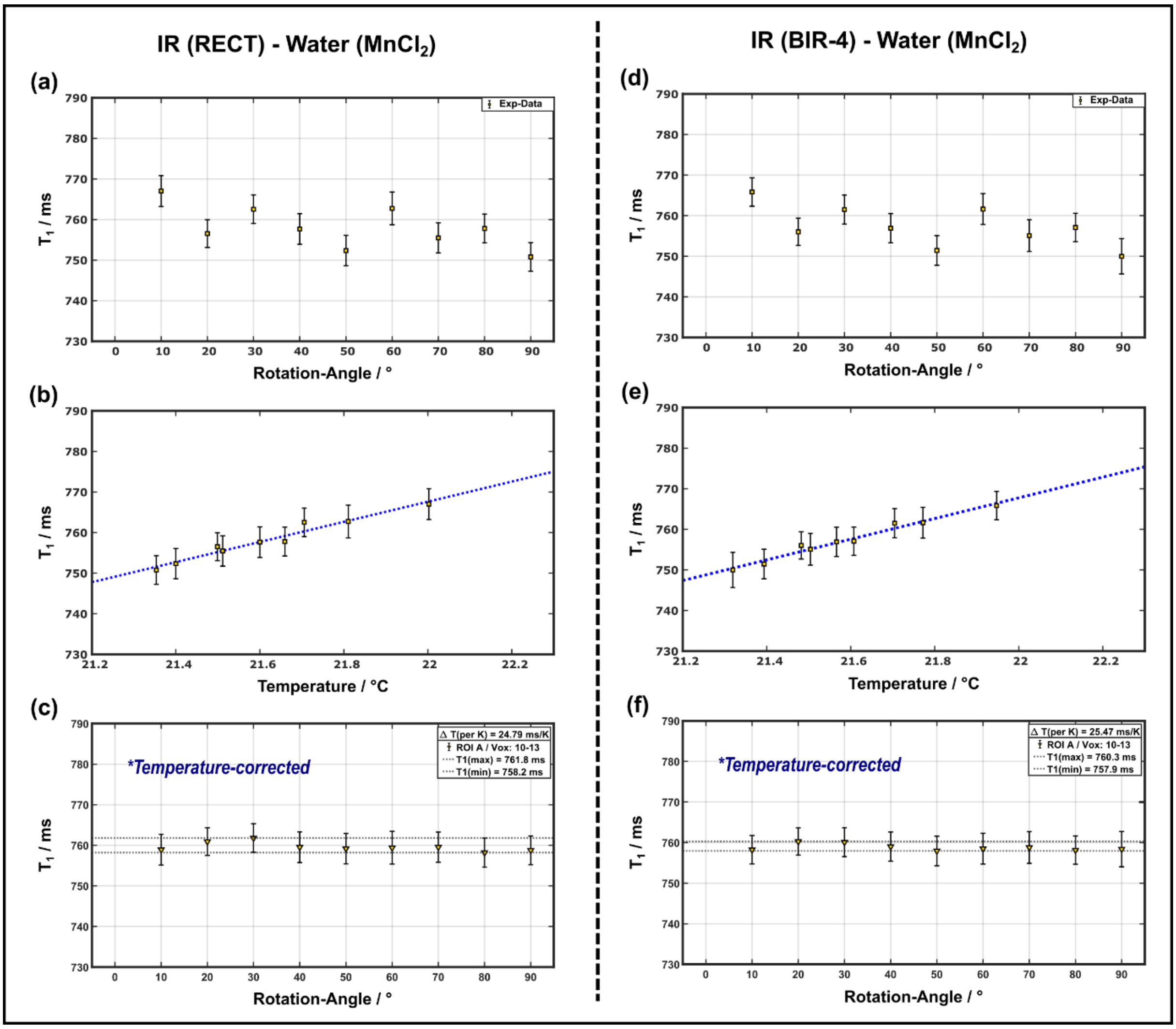

Supplementary Figure S5. Spin-lattice relaxation times measured as a function of the rotation angle in the doped water (doped with $MnCl_2$) sample at room temperature with IR and monoexponential fitting. **(a–c)** Experiments with inversion by a 40μs hard pulse, **(d–f)** experiments with a BIR-4 adiabatic inversion pulse. Error bars indicate SDs over the ROI (consisting of 4 consecutive voxels as in the porcine WM sample). After temperature correction, obtained from the T_1 drift with the measured room temperature (approx. 25 ms/K), stable T_1 estimates (variations <0.5%) were obtained with both IR acquisitions.

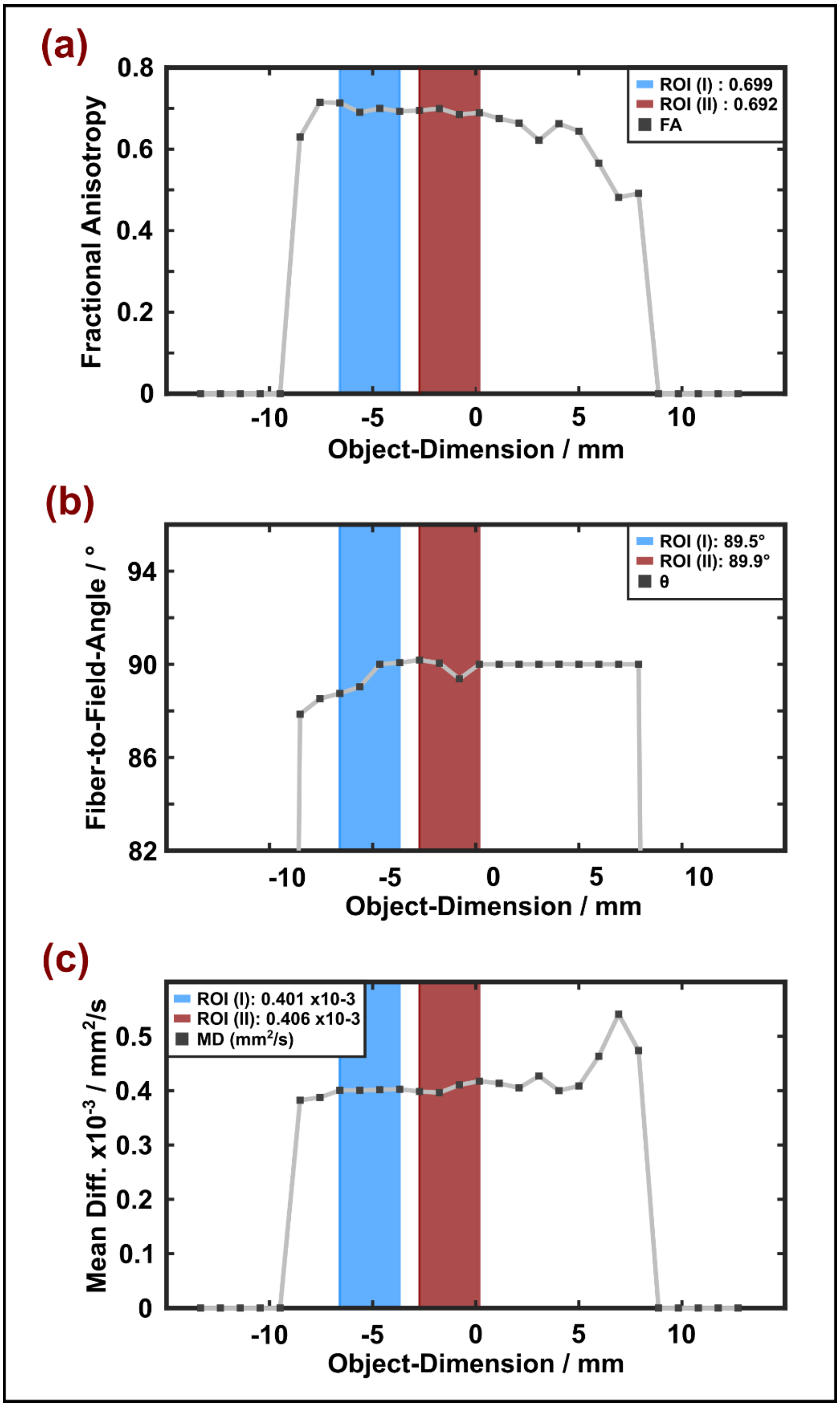

Supplementary Figure S6. Results of 1D DTI at 36 °C with 60 diffusion-sensitizing gradient directions and the sample aligned with the magnet's physical y-direction (i.e., at 90° relative to $\mathbf{B}_0$). Shaded areas indicate the two regions selected for analysis. Both are characterized by similarly high FA values **(a)**. The alignment of the main fiber direction with the axis of to the NMR tube is reflected by very small deviations (<2°) between θ_{FB} obtained from the DTI and the adjusted rotation angle **(b)**. Similar results were obtained in experiments performed at 22 °C (see Figure S6). As expected, the mean diffusivity was reduced by about 25% at room temperature **(c)**.

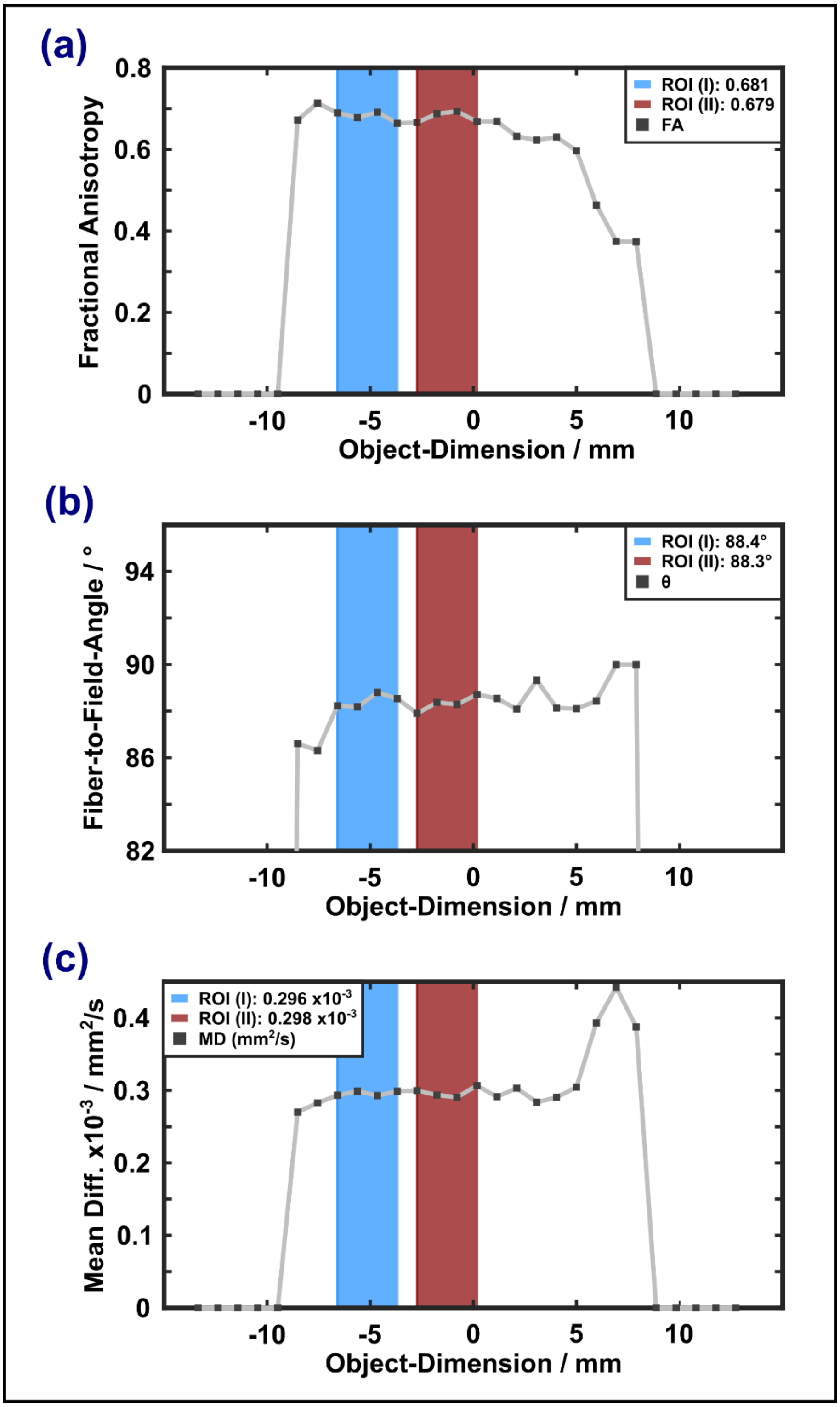

Supplementary Figure S7. Results of 1D DTI at 22 °C with 60 diffusion-sensitizing gradient directions and the sample aligned with the magnet's physical y-direction (i.e., at 90° relative to $\mathbf{B}_0$). Shaded areas indicate the two regions selected for analysis. Both are characterized by similarly high FA values **(a)**. The alignment of the main fiber direction with the axis of to the NMR tube is reflected by very small deviations (<2°) between θ_{FB} obtained from the DTI and the adjusted rotation angle **(b)**. The mean diffusivity was reduced by about 25% compared to the experiment at 36 °C (see Figure S5C) **(c)**.

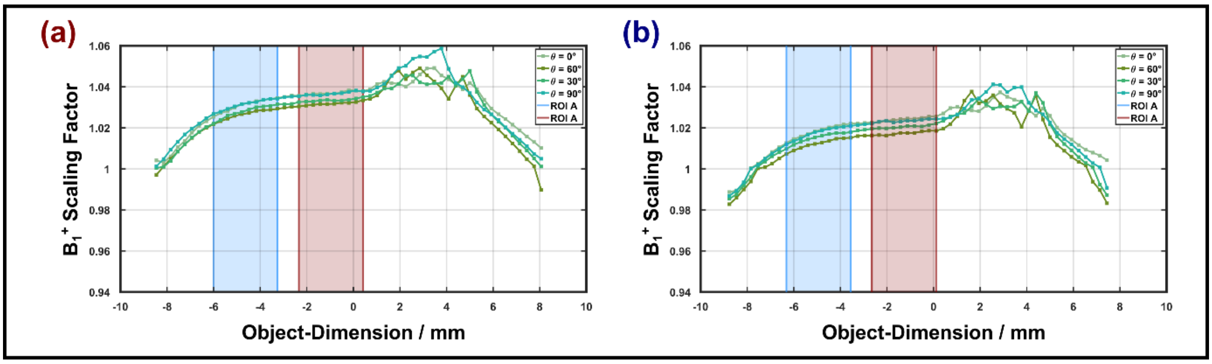

Supplementary Figure S8. 1D transmit-field profiles (expressed as a B_1^+ scaling factor for the nominal flip angle) along the 5mm NMR tube (nominal resolution 0.306 mm) filled with the spinal cord sample, measured at different orientations of the coil plus sample relative to $\mathbf{B}_0$ at 36 °C **(a)** and at 22 °C **(b)**. A subtle overall shift of the mean B_1^+ is visible in comparisons of different rotation angles, however, with negligible magnitude (<1%) compared to the position-dependent profile along the sample axis. The general shape of the profiles was almost independent of the rotation angle. The ‘noisy region’ in the (positive) region around 2–4 mm is caused by a low signal amplitude in this area (and, hence, less reliable flip-angle estimates), which and is not considered in further analyses. The blue and red shaded areas correspond to the two ROIs selected for the final analysis of the T_1 and MT experiments (see Figures S5 and S6)

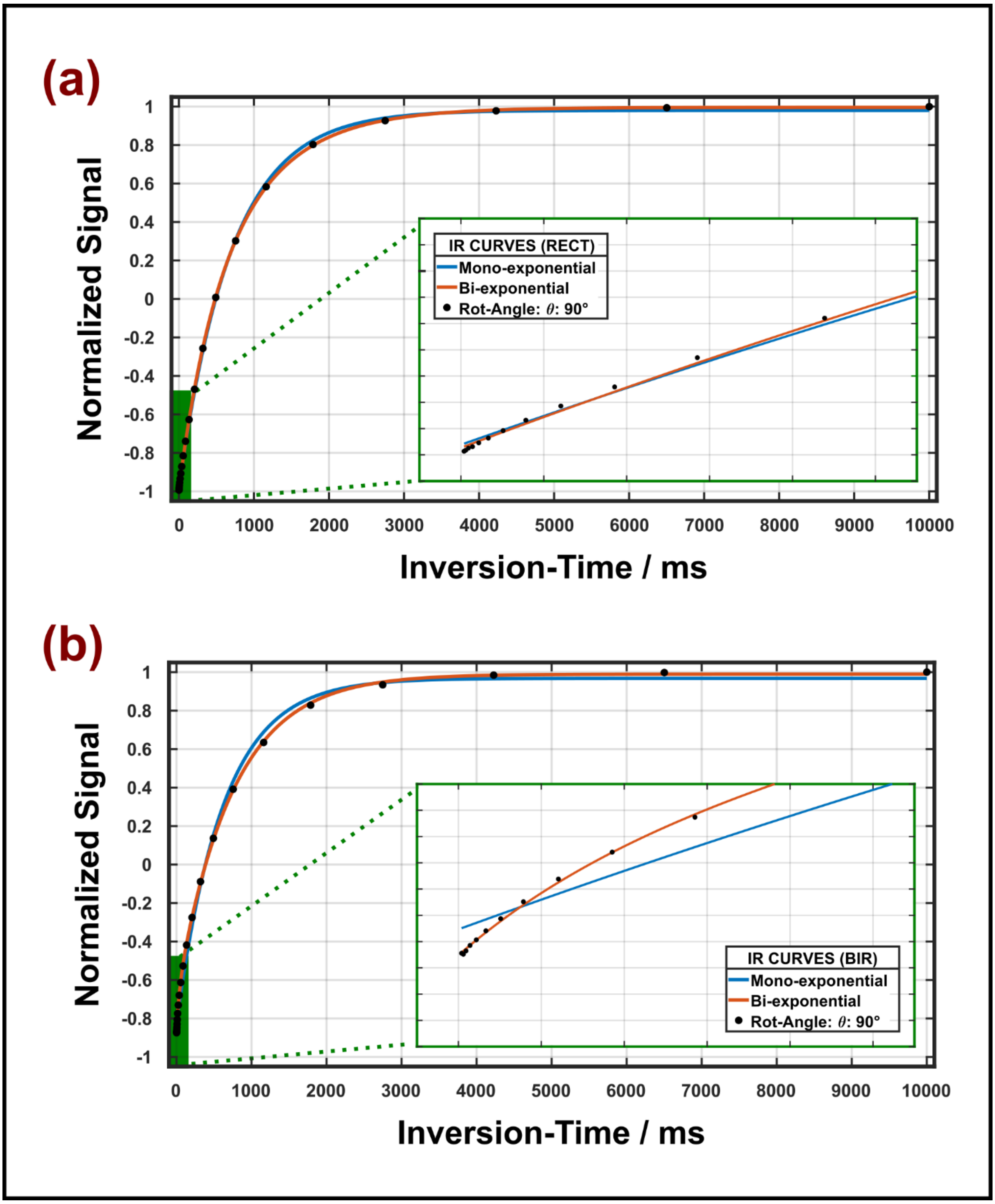

Supplementary Figure S9. Inversion-recovery curves for inversion by a 40-µs rectangular **(a)** and a 5-ms adiabatic pulse **(b)** at 36 °C and a rotation angle of 90°. The blue and orange lines show results from monoexponential and biexponential fitting, respectively. Inserts show a magnified region with data measured at TI<100 ms (green area). There is a deviation from monoexponential recovery, especially in the experiment with the longer adiabatic inversion pulse.

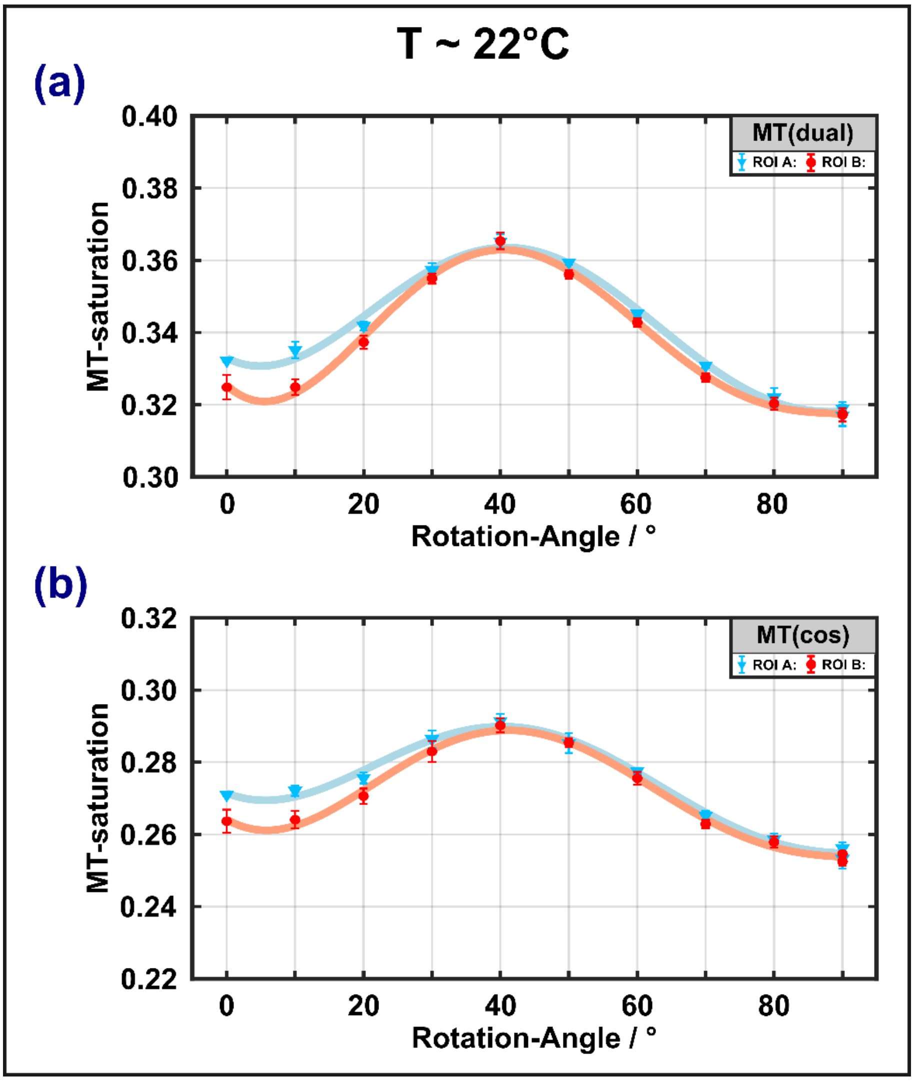

Supplementary Figure S10. MT-saturation obtained at 22 °C with off-resonant irradiation at an offset frequency of ±10 kHz with $\gamma B_{1,\mathrm{RMS}}^{+}/(2\pi)$ = 500 Hz **(a,b)**. Dual-sided saturation was achieved by either alternating the offset-frequency **(a)** or cosine modulation **(b)** of the Gaussian MT pulse (symbols, lines and ROI definitions as in Fig 2). An overall identical orientation dependence with a maximum at θ_{FB} between 30° and 40° is obtained with minor differences in the range of MT_{sat} variation (3-4% and 2-3% for frequency alternation and cosine modulation, respectively.

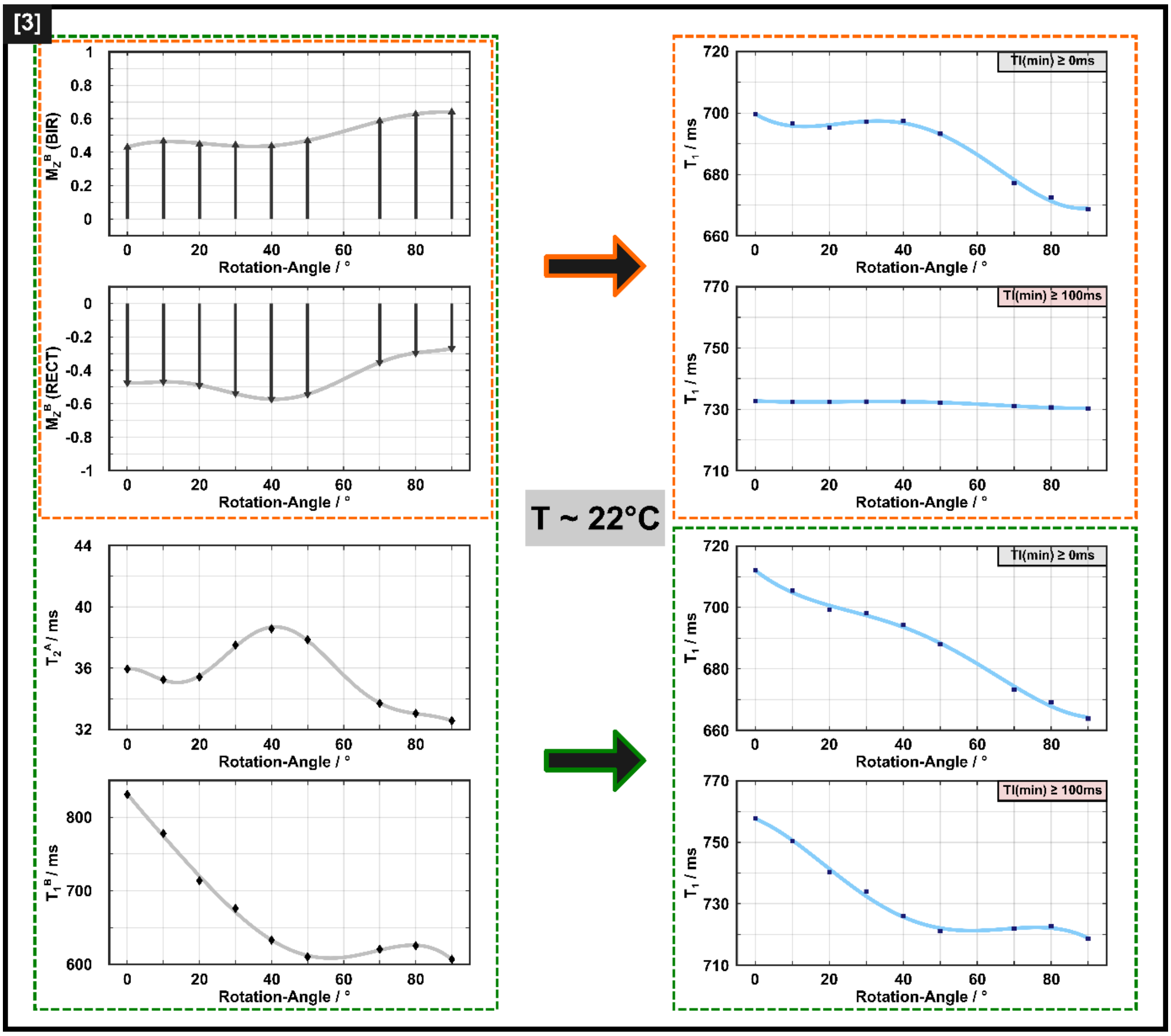

Supplementary Figure S11. Overview of anisotropic BSB model parameters as a function of θ_{FB} (left column) at 22 °C, and resulting orientation-dependent T_1 (right column) from fits to the IR data. The additional θ_{FB}-independent BSB parameters were MPF = 0.16, T_1^A = 750 ms, and k = 16.7 s^{-1}. Solid lines are guides to the eye. The illustrations included in frames with orange broken lines show the orientation dependence of $M_z^B(0^+)$ according to model *(1)*, with partial saturation obtained with the 5ms BIR-4 pulse and partial inversion obtained with the 40μs hard pulse. To better visualize the differences in $M_z^B(0^+)$, grey arrows indicate that $M_z^B(0^+)$ after the BIR-4 pulse still points along the external magnetic field, whereas it is partially inverted after application of the RECT pulse. This model yields orientation-dependent T_1 if all TIs are included in the monoexponential fitting. However, it leads to almost invariant T_1 upon restricting the analysis to TI>100 ms, which is inconsistent with the experimental data in Figure 2. The trends in the anisotropy of T_1 estimates obtained with both all TIs or only those >100 ms could be reproduced by assuming additional orientation dependencies of T_2^A and T_1^B (illustrations in frames with the green broken lines).

References

1 Müller, D. K., Pampel, A. & Möller, H. E. Matrix-algebra-based calculations of the time evolution of the binary spin-bath model for magnetization transfer. J. Magn. Reson. 230, 88-97 (2013). https://doi.org/10.1016/j.jmr.2013.01.013

2 Henkelman, R. M. et al. Quantitative Interpretation of magnetization-transfer. Magn. Reson. Med. 29, 759-766 (1993). https://doi.org/10.1002/mrm.1910290607

3 McConnell, H. M. Reaction rates by nuclear magnetic resonance. J. Chem. Phys. 28, 430-431 (1958). https://doi.org/10.1063/1.1744152

4 Manning, A. P., MacKay, A. L. & Michal, C. A. Understanding aqueous and non-aqueous proton T1 relaxation in brain. J. Magn. Reson. 323 (2021). https://doi.org/10.1016/j.jmr.2020.106909

5 Graham, S. J. & Henkelman, R. M. Understanding pulsed magnetization transfer. J. Magn. Reson. Imaging 7, 903-912 (1997). https://doi.org/10.1002/jmri.1880070520

6 Morrison, C. & Henkelman, R. M. A Model for magnetization-transfer in tissues. Magn. Reson. Med. 33, 475-482 (1995). https://doi.org/10.1002/mrm.1910330404

7 Wennerström, H. Proton nuclear magnetic resonance lineshapes in lamellar liquid crystals. Chem. Phys. Lett. 18, 41-44 (1973). https://doi.org/10.1016/0009-2614(73)80333-1

8 Pampel, A., Müller, D. K., Anwander, A., Marschner, H. & Möller, H. E. Orientation dependence of magnetization transfer parameters in human white matter. NeuroImage 114, 136-146 (2015). https://doi.org/10.1016/j.neuroimage.2015.03.068

9 Murase, K. & Tanki, N. Numerical solutions to the time-dependent Bloch equations revisited. Magn. Reson. Imaging 29, 126-131 (2011). https://doi.org/https://doi.org/10.1016/j.mri.2010.07.003

10 Moler, C. & Van Loan, C. Nineteen dubious ways to compute the exponential of a matrix, twenty-five years later. SIAM Rev. 45, 3-49 (2003). https://doi.org/10.1137/S00361445024180
